# Optimizing rice yield, quality and nutrient use efficiency through combined application of nitrogen and potassium

**DOI:** 10.3389/fpls.2024.1335744

**Published:** 2024-03-11

**Authors:** Guangyi Chen, Qiang Duan, Chaoyue Wu, Xingmei He, Mingming Hu, Congmei Li, Yuyuan Ouyang, Ligong Peng, Hong Yang, Qiuqiu Zhang, Qinggui Jiang, Yan Lan, Tian Li

**Affiliations:** ^1^ College of Agronomy, Sichuan Agricultural University, Chengdu, China; ^2^ Station of Seed Management, Agricultural and Rural Bureau of Xuanhan County, Dazhou, China; ^3^ Rice Research Institute, Sichuan Agricultural University, Chengdu, China; ^4^ Crop Research Institute, Sichuan Academy of Agricultural Sciences, Chengdu, China; ^5^ Station of Foundation and Improved Seed-breeding, Sichuan Provincial Department of Agriculture and Rural Affairs, Chengdu, China; ^6^ College of Life Science and Engineering, Southwest University of Science and Technology, Mianyang, China

**Keywords:** rice, combined application of nitrogen and potassium, nutrient absorption-utilization, yield, quality

## Abstract

Reasonable nitrogen (N) and potassium (K) application rates can effectively improve fertilizer use efficiency, rice yield and quality. A two-year field experiment was conducted with combined application of three N rates (135, 180, and 225 kg ha^-1^, denoted as N1-N3) and four K rates (0, 90, 135, and 180 kg ha^-1^, denoted as K0-K3) using super indica hybrid rice cultivar Yixiangyou (YXY) 2115 to explore the effects of co-application of N and K on rice growth and development. The results indicated that the combined application of N and K had significantly interactive effects on dry matter (DM) accumulation, nutrients absorption, N harvest index (NHI), K harvest index (KHI), spikelets per panicle and most rice quality indexes. The highest total DM accumulation (17998.17-19432.47 kg ha^-1^) at maturity stage was obtained under N3K2. The effect of co-application of N and K on nutrients absorption and utilization varied between the two years and within each year. The highest total N and K accumulations at maturity stage were observed under N3K1 and N3K2, respectively, while the highest N recovery efficiency (NRE) and K recovery efficiency (KRE) were observed under N1K3. High expression levels of N and K metabolism-related genes in rice grains were observed under N3K2 or N3K3, consistent with N and K uptake. Co-application of N and K increased rice yield significantly and the highest yield (6745.02-7010.27 kg ha^-1^) was obtained under N2K2. As more N was gradually applied, rice appearance quality improved but milling, cooking and eating quality decreased. Although appropriate application of K could improve rice milling, cooking and eating quality, it reduced appearance quality. The optimal milling, cooking and eating quality were obtained under N1K2, while the best appearance quality was obtained under N3K0. Overall, a combination of 135-210 kg ha^-1^ N and 115-137 kg ha^-1^ K application rates was recommended for achieving relatively higher yield and better quality in rice production.

## 1 Introduction

Rice (*Oryza sativa* L.) stands as a primary global food, with China playing a prominent role as both a major producer and consumer of this essential crop. Over half of China’s population relies on rice as their staple food. Consequently, the advancement of rice production holds paramount significance in meeting the living needs of population and ensuring food security (Karthika et al., 2023). Nowadays, fertilizers have evolved into the principle source of soil nutrient provision. The enhancement of crop yield is inseparable from the application of chemical fertilizers, no exception for rice production. Approximately 30-50% of global increase in crop yield can be attributed to fertilizer input (Erisman et al., 2008), while the increase rate for rice yield can attain a notable 57.4% (Wang et al., 2010a).

N and K are essential nutrients for rice growth and development, and play important roles in the determination of both yield and quality. The amount of N input in rice production in China significantly surpasses the global average level (Ludemann et al., 2022). However, there is a discernible decline in the utilization rate of N, possibly attributed to the persistent escalation of N application rates (Ju et al., 2015). The application rate of N in rice production in southwest China reaches approximately 180 kg ha^-1^, yet the absorption and utilization rate of N hovers between 32-37% (Zhang et al., 2015). However, due to the more apparent immediate effects of N fertilizer application, it is a prominent problem that the preference for higher N inputs over the combined application of N and K in Chinese rice production (Li, 2017). In contrast to N, the application of K in rice production is often insufficient. Rice is a crop that requires a high amount of K, with an uptake ranging from 150-300 kg ha^-1^ per season, comparable to or even exceeding the N uptake (Xu et al., 2015; Zhu et al., 2019). Improper application of fertilizer will lead to the diminished nutrients utilization efficiency, yield reduction, deterioration of rice quality and environmental pollution, which may have the potential to impact human living condition and the sustainable development of agriculture and ecology (Li et al., 2018; Zhang et al., 2019). Hence, the efficiency of fertilizer absorption and utilization after application, as well as optimization of fertilization methods, should be taken into consideration to mitigate the adverse impacts associated with fertilization.

The hybrid indica rice cultivar Yixiangyou 2115 is currently extensively cultivated in southwest China, acclaimed for its high quality, substantial yield and resistance to multiple stresses. It was identified as “Super Rice” in 2015 by the Ministry of Agriculture. So far, it has been widely promoted and applied for more than 1.3 million hectares and become a representative cultivar of hybrid rice in China. However, limited studies can be found on the cultivation techniques aimed at achieving both high yield and high quality for this specific rice cultivar. N and K have certain regulatory effects on rice growth and development. The rational application of N and K can significantly enhance fertilizer utilization efficiency, thereby positively influencing both rice yield and quality. The phenomenon of heavy application of N with a comparably lighter application of K is common due to the relative lower cost of N fertilizer and the established fertilization habits, which leads to high yield but low efficiency in rice production. Numerous studies have explored the individual effects of N or K on rice growth and development. A comprehensive understanding of the combined effects of N and K on nutrient absorption, utilization, as well as their impact on rice yield and quality, remains an area requiring further systematic investigation. Therefore, field experiments were conducted with combined application of three N rates (135, 180, and 225 kg ha^-1^) and four K rates (0, 90, 135, and 180 kg ha^-1^) using super indica hybrid rice cultivar YXY 2115 to explore the interactive effects of N and K on rice nutrients absorption and utilization, yield and quality. The objective of this study was to clarify the optimal ration of N and K conducive to achieving both high yield and high quality for YXY 2115. Also, the expression levels of N and K metabolism-related key genes *OsNRT1.1B*, *OsAMT2;1*, *OsNR2*, *OsHAK1*, *OsHAK5* and *OsAKT1* were quantified to assess the molecular response of rice grains to different fertilizer application rates. This study was expected to provide a theoretical basis for the optimization and improvement of cultivation methods to achieve high yield, high quality, and reduce fertilizer input for rice production in southwest China, as well as the large-scale promotion and application of this cultivar.

## 2 Materials and methods

### 2.1 Experimental site and materials

**Figure 1:**
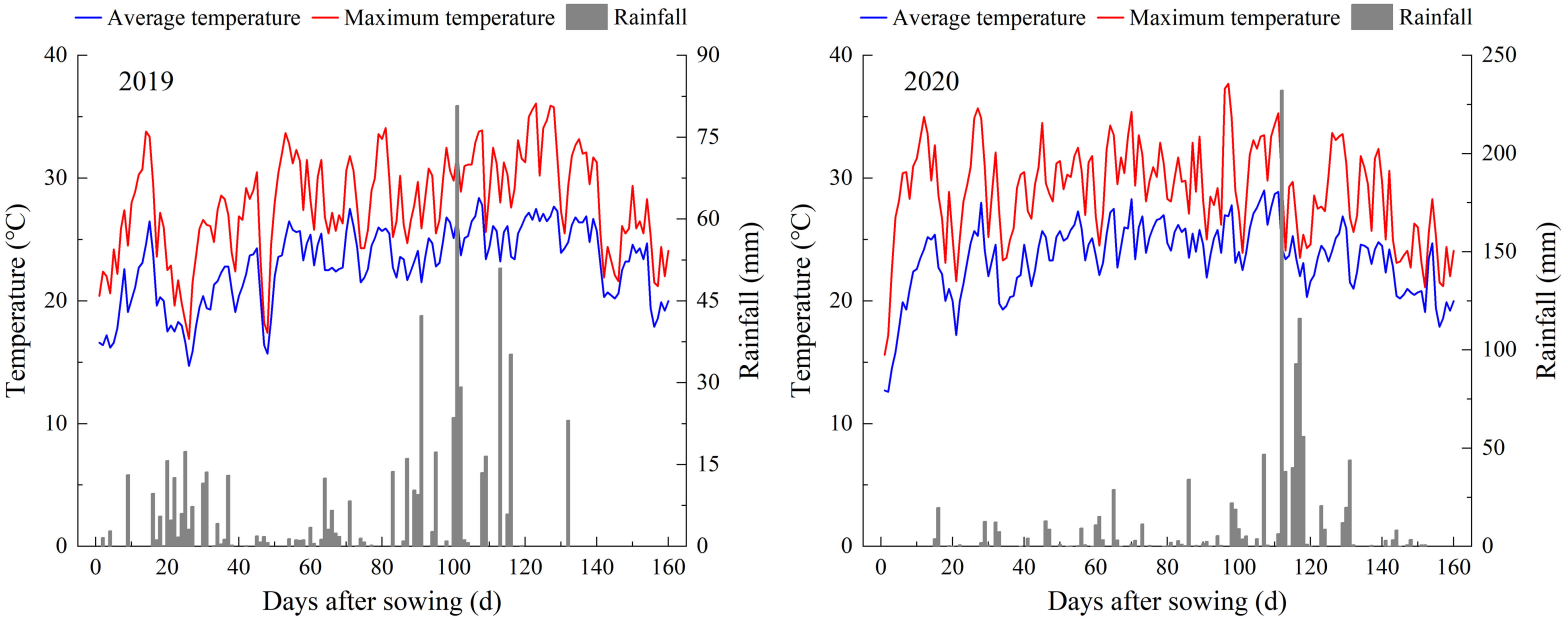
Climate data during the experimental periods.

Field experiments were conducted during the growing seasons of 2019 and 2020 at the research farm of Sichuan Agricultural University, Wenjiang city, Sichuan Province, China (30°70′ N, 103°83′ E). The soil of the plot was clay soil. Prior to the establishment of the field experiment, soil samples from the topsoil layer (0-0.20 m) were analyzed. The climate data and analysis results of the top soil layer were shown in **Figure 1** and **Table 1**, respectively. During rice growth period in two years, the daily average temperature and rainfall were 22.78 °C and 3.69 mm in 2019, and 23.31 °C and 6.53 mm in 2020, respectively. Super indica hybrid rice cultivar Yixiangyou 2115 (Sichuan Agricultural University), which is widely cultivated in southwest China with high yield and quality with the whole growth period of 146 days, was chosen and used as the test material.

**Table 1:** Soil properties of the top soil layer (0-0.20 m) at the experimental sites.

Years	pH	Organic matter (g·kg^-1^)	Total N content (g·kg^-1^)	Total P content (g·kg^-1^)	Total K content (g·kg^-1^)	Available N (mg·kg^-1^)	Available P (mg·kg^-1^)	Available K (mg·kg^-1^)
2019	6.90	27.32	1.81	0.75	10.13	97.16	24.30	77.08
2020	6.39	15.97	1.86	1.11	18.07	86.54	21.68	76.75

N, P, K represent nitrogen, phosphorus and potassium, respectively.

### 2.2 Experimental design

Two factor factorial field trials with three levels of N and four levels of K were conducted in two years, using a randomized complete block design with three replicates. Twelve treatments were established by the complete combination of three N application rates (135, 180 and 225 kg ha^-1^, denoted as N1, N2 and N3, respectively) and four K application rates (0, 90, 135 and 180 kg ha^-1^, denoted as K0, K1, K2 and K3, respectively). No nitrogen application treatment was established and used to calculate the nutrients absorption and utilization only.

Seeds were sown on 12 April 2019 and 14 April 2020, and the seedlings were transplanted on 27 May 2019 and 26 May 2020, and rice was harvested on 19 Sept 2019 and 20 Sept 2020, respectively. The area of each test plot was 5.0 m×5.0 m, and the transplant density was 30 cm×25 cm (row spacing × plant spacing) with one seedling per hill. Urea (N, 46.4%) was used as the N source, superphosphate (P_2_O_5_, 12.0%) was used as the phosphorus (P) source, and potassium chloride (K_2_O, 60.0%) was used as the K source. N fertilizer was used as basal manure and top dressing (twenty days after transplanting) at a 6:4 ratio. Basal N, P (90 kg ha^-1^) and K were applied to the soil one day before transplanting. For the fertilizer treatments, ridges with plastic film were used for separation, and protection lines were established between the treatment blocks to ensure the isolation of the experimental plots. During the whole growing season, rice was continuously flooded with the river water. Field management, including the prevention and control of pests and weeds, was conducted according to the local cultural practices.

### 2.3 Measurements and methods

#### 2.3.1 Dry matter accumulation

Ten representative hills were sampled from each plot based on the average number of tillers at full heading stage (9 Aug 2019 and 13 Aug 2020) and maturity stage (14 Sept 2019 and 15 Sept 2020), respectively. Samples were then separated into different parts, and were oven-dried at 105°C for 30 min and then to constant weight at 80°C to determine the dry weight. DM of stem-sheath, leaf, panicle, and total biomass were recorded.

#### 2.3.2 Absorption and utilization efficiency of N and K

After weighted, all dried samples were ground to make powder, passed through a 0.20 mm sieve, and digested with a mixture of concentrated H_2_SO_4_ and H_2_O_2_ to determine N and K concentrations. The N concentration was determined using a continuous flow analyzer (AA3, Seal Analytical Inc., Southampton, UK) while K was determined using flame photometer (M-410, Sherwood Scientific Ltd., Cambridge, UK). The nutrients absorption and utilization efficiency were analyzed as below (Wang et al., 2004, 2009).


$$\text{Plant N }\left(\text{K}\right)\text{ accumulation amount}\;(\text{kg }{\text{ha}}^{-1})=\text{Plant N }\left(\text{K}\right)\text{ concentration}\times \text{Plant DM weight}$$

$$\begin{array}{c} \text{Total N }\left(\text{K}\right)\text{ accumulation amount }\left(\text{kg }{\text{ha}}^{-1}\right)=\\ \text{The sum of N }\left(\text{K}\right)\text{ accumulations in the aboveground parts}\;\left(\text{stem-sheath, leaf and panicle}\right) \end{array}$$

$$\begin{array}{c} \text{N }\left(\text{K}\right)\text{ recovery efficiency }\left(\text{NRE and KRE},\%\right)\\ =(\text{Total plant N }\left(\text{K}\right)\text{ accumulation amount of N }\left(\text{K}\right)-\text{fertilized plot}\\ -\text{Total plant N }\left(\text{K}\right)\text{ accumulation amount of zero}-\text{N }\left(\text{K}\right)\text{ plot})\\ /\text{Applied N }\left(\text{K}\right)\text{ rate of fertilized plot}\times 100\% \end{array}$$

$$\begin{array}{c} \text{N }\left(\text{K}\right)\text{ agronomic efficiency }\left(\text{NAE and KAE, kg }{\text{kg}}^{-1}\right)=\\ \left(\text{Rice yield of N }\left(\text{K}\right)-\text{fertilized plot}-\text{rice yield of zero}-\text{N }\left(\text{K}\right)\text{ plot}\right)\\ /\text{Applied N }\left(\text{K}\right)\text{ rate of fertilized plot} \end{array}$$

$$\begin{array}{c} \text{N }\left(\text{K}\right)\text{ contribution rate }\left(\text{NCR and KCR,}\%\right)=\\ \left(\text{Rice yield of N }\left(\text{K}\right)-\text{fertilized plot}–\text{rice yield of zero}-\text{N }\left(\text{K}\right)\text{ plot}\right)\\ /\text{Rice yield of N }\left(\text{K}\right)-\text{fertilized plot}\times 100\% \end{array}$$

$$\begin{array}{c} \text{N }\left(\text{K}\right)\text{ harvest index }\left(\text{NHI and KHI,}\%\right)=\\ \text{N }\left(\text{K}\right)\text{ accumulation in panicle at maturity stage}/\text{Total N }\left(\text{K}\right)\\ \text{ accumulation in the aboveground parts}\left(\text{stem-sheath, leaf and panicle}\right)\times 100\% \end{array}$$

#### 2.3.3 RNA isolation and RT-qPCR

Total RNA samples were obtained from rice grains at mid-filling stage (15 days after flowering) using RNA Trizol reagent (Invitrogen, Carlsbad, CA, USA) and reverse-transcribed into cDNA using Revertase Transcription kit (Nanjing Vazyme Medical Technology Co., Ltd., Nanjing, China). The products were quantified using a real-time PCR detection system, following the manufacturer’s instructions (SYBR Green Master Mix, Vazyme). The rice Actin gene was used as an internal control. Treatment without nitrogen fertilizer was used as CK. The PCR primers used were listed in **Supplementary Table S5**.

#### 2.3.4 Yield and yield components

Rice was harvested at maturity stage and the yield in each experimental plot was recorded after measuring moisture content and removing impurities. Grain yield was adjusted to a moisture content of 13.5%. The number of effective tillers per hill was determined before harvest using 30 plants per plot. A total of 10 selected plants were separated into single tillers according to the marked date, and were used to measure 1000-grain weight, seed-setting rate, and filled grain number per panicle.

#### 2.3.5 Rice quality

At harvest, 10 plants from each plot were sampled randomly and allowed to dry naturally in the sun to assess rice milling quality, appearance quality, eating quality and rapid visco-analyzer (RVA) value after the material was stored at room temperature for 3 months.

##### 2.3.5.1 Milling quality

About 100.0 g rice grains were processed using a rice huller (JLG-2118, Taizhou Food Instrument Co., Ltd., Zhejiang, China) to obtain brown rice. The brown rice was polished using a rice milling machine (JNMJ-3, Taizhou Food Instrument Co., Ltd., Zhejiang, China) to obtain milled rice. In order to obtain head rice, grain with a length longer than 3/4 of its total length was separated from the milled rice using a broken rice separator (FQS-13X20, Taizhou Food Instrument Co., Ltd., Zhejiang, China). The brown rice, milled rice, and head rice are expressed as percentages of the total grain weight.

##### 2.3.5.2 Appearance quality

The grain length, width, length/width, chalkiness rate and chalkiness degree were determined using a grain appearance analyzer (JMWT12, Dongfu Jiuheng Instrument Technology Co., Ltd., Beijing, China).

##### 2.3.5.3 Rapid visco-analyzer value

A 3.00 g sample and 25.0 mL of distilled water were added to a test tube. Pasting properties were measured using an RVA device (3-D, Newport Scientific, Sydney, Australia) and analyzed with Thermal Cycle for Windows software. Viscosity values were measured in a rapid viscosity analyzer unit (RVU).

##### 2.3.5.4 Eating quality

The sensory properties of the cooked rice were measured using a rice sensory analyzer (STA 1B, Satake Asia Co., Ltd., Tokyo, Japan). Milled rice (30.00 g) was washed in a stainless-steel container and then transferred into a 50 mL aluminum box containing 40 mL of water. The milled rice was cooked in a multifunctional, timed food steamer (GF-339, Goodway Electrical Enterprise Ltd., Hong Kong, China). After the cooking, the sensory properties of the cooked rice were determined. Cooked rice texture properties were measured using a rice texture analyzer (RHS 1A, Satake Asia Co., Ltd., Tokyo, Japan).

### 2.4 Statistical analysis

Data were analyzed by using analysis of variance (ANOVA), and means were compared based on the least significant difference (LSD) test at the 0.05 probability level by using SPSS 25.0 (Statistical Product and Service Solutions Inc., Chicago, IL, USA). The assumptions of variance analysis were tested by ensuring that the residuals were random, homogenous, with a normal distribution about/above a mean of zero (Ali et al., 2013; Abolfazl et al., 2017). Origin Pro 2020 (OriginLab, Northampton, MA, USA) was used to draw the figures. Matlab (Mathworks, Natick, MA, USA) was used to establish the binary quadratic fitting equation and figures.

## 3 Results

### 3.1 Dry matter accumulation

**Figure 2:**
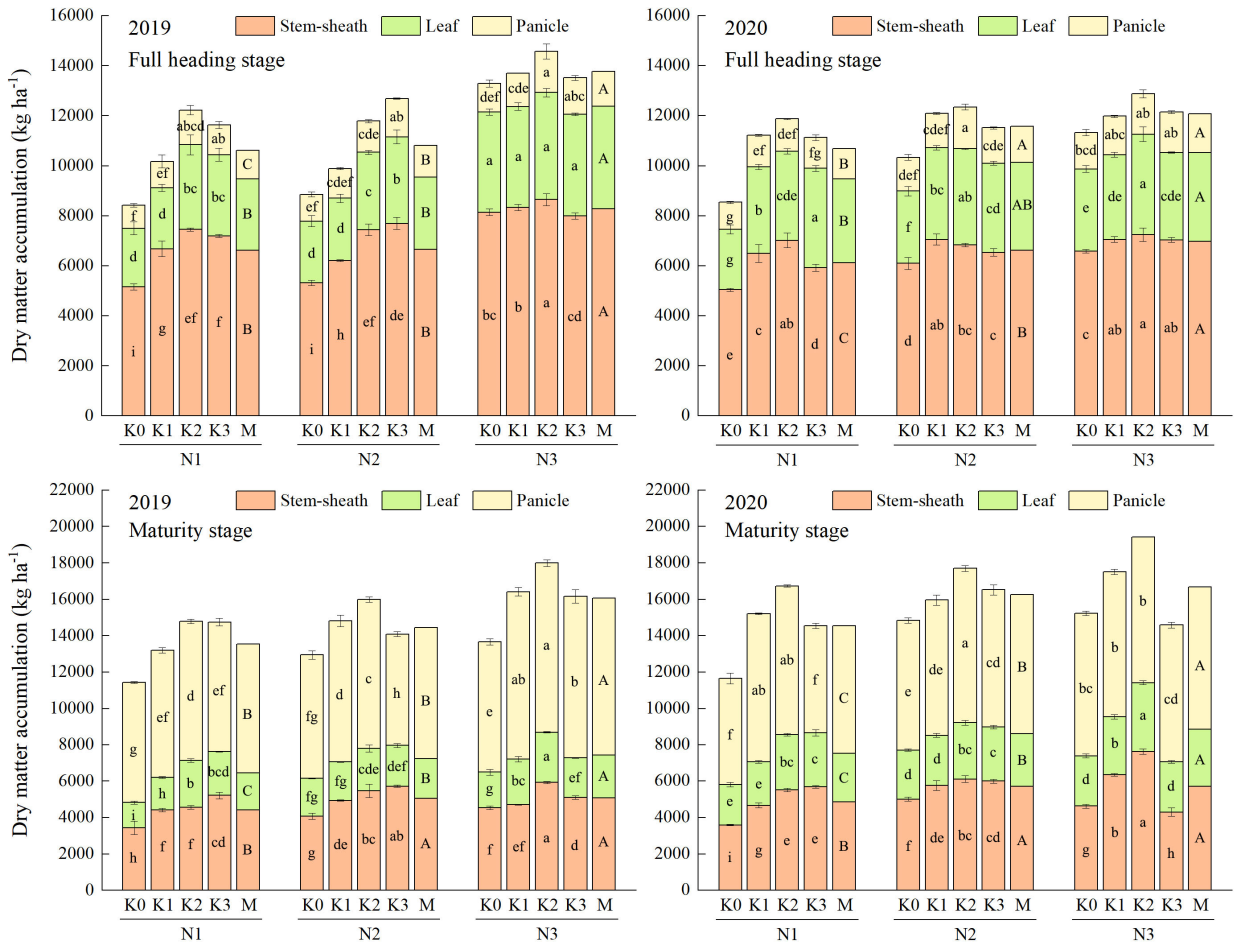
Effects of combined application of N and K on dry matter accumulation of YXY 2115 at full heading stage (above) and maturity stage (below). K0, K1, K2 and K3 refer to the different potassium fertilizer treatments (0, 90, 135 and 180 kg ha^-1^, respectively). N1, N2 and N3 refer to the different nitrogen fertilizer treatments (135, 180 and 225 kg ha^-1^, respectively). M represents the average of different potassium fertilizer levels under the same nitrogen fertilizer level. Different lowercase (uppercase) letters in the same color column mean the significant difference between treatments at *p* < 0.05. The data presented are the mean ± standard deviation, *n* = 3.

DM accumulation in the aboveground organs of rice could be enhanced by increasing N application, and could be further increased by applying appropriate K application at high N level (**Figure 2**). The maximum values of DM accumulation in stem-sheath, leaf and panicle were observed under N3 at both full heading stage and maturity stage. Compared with other treatments, the maximum increases of total DM accumulation were obtained under N3K2, which were 57.54-66.62% at maturity stage. Variance analysis showed that co-application of N and K had extremely significant interactive effects on the DM accumulation in the aboveground organs of rice (except panicle at full heading stage) (**Supplementary Table S1**).

### 3.2 Uptake of N and K

**Figure 3:**
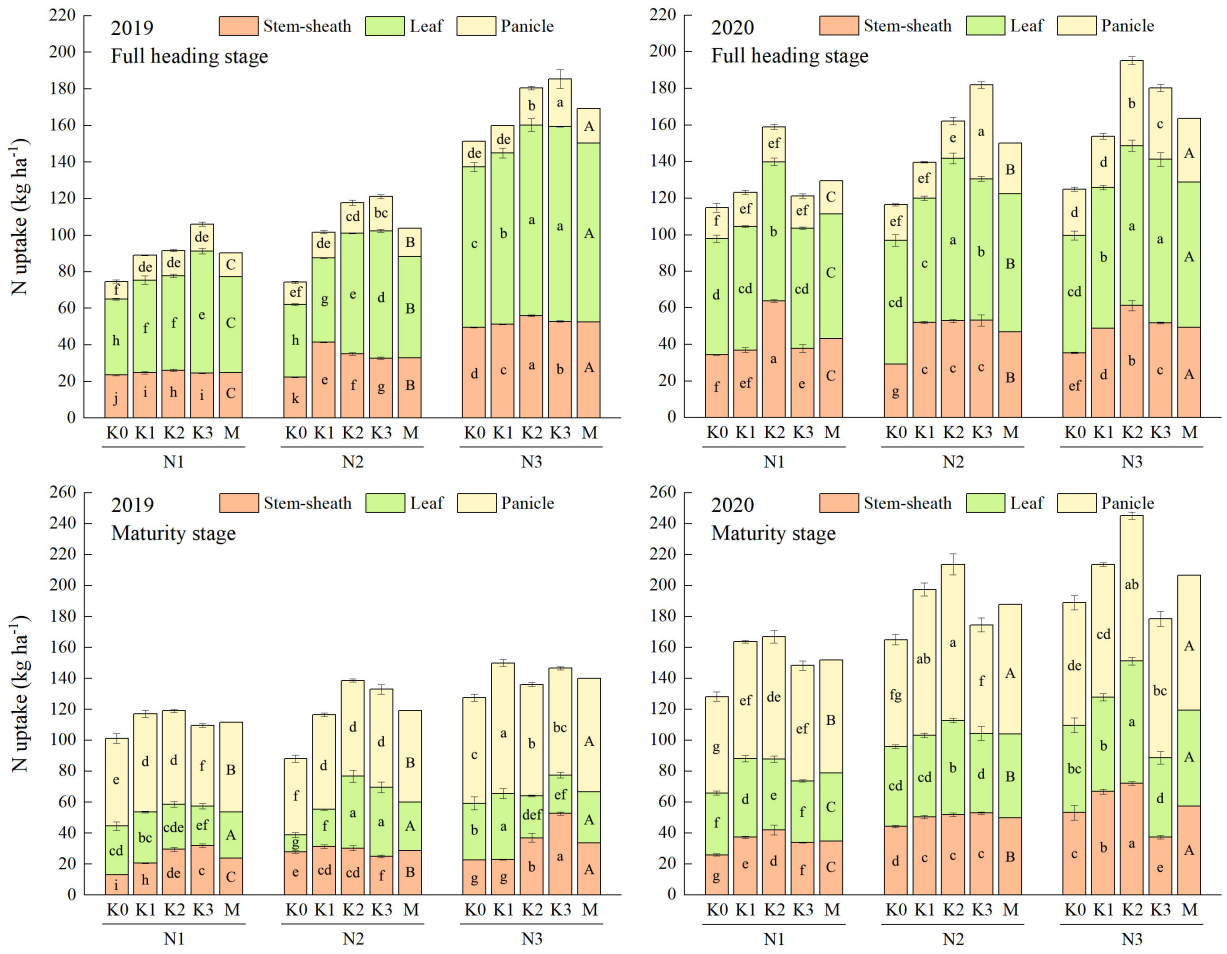
Effects of combined application of N and K on N uptake of YXY 2115 at full heading stage (above) and maturity stage (below). K0, K1, K2 and K3 refer to the different potassium fertilizer treatments (0, 90, 135 and 180 kg ha^-1^, respectively). N1, N2 and N3 refer to the different nitrogen fertilizer treatments (135, 180 and 225 kg ha^-1^, respectively). M represents the average of different potassium fertilizer levels under the same nitrogen fertilizer level. Different lowercase (uppercase) letters in the same color column mean the significant difference between treatments at *p* < 0.05. The data presented are the mean ± standard deviation, *n* = 3.

**Figure 4:**
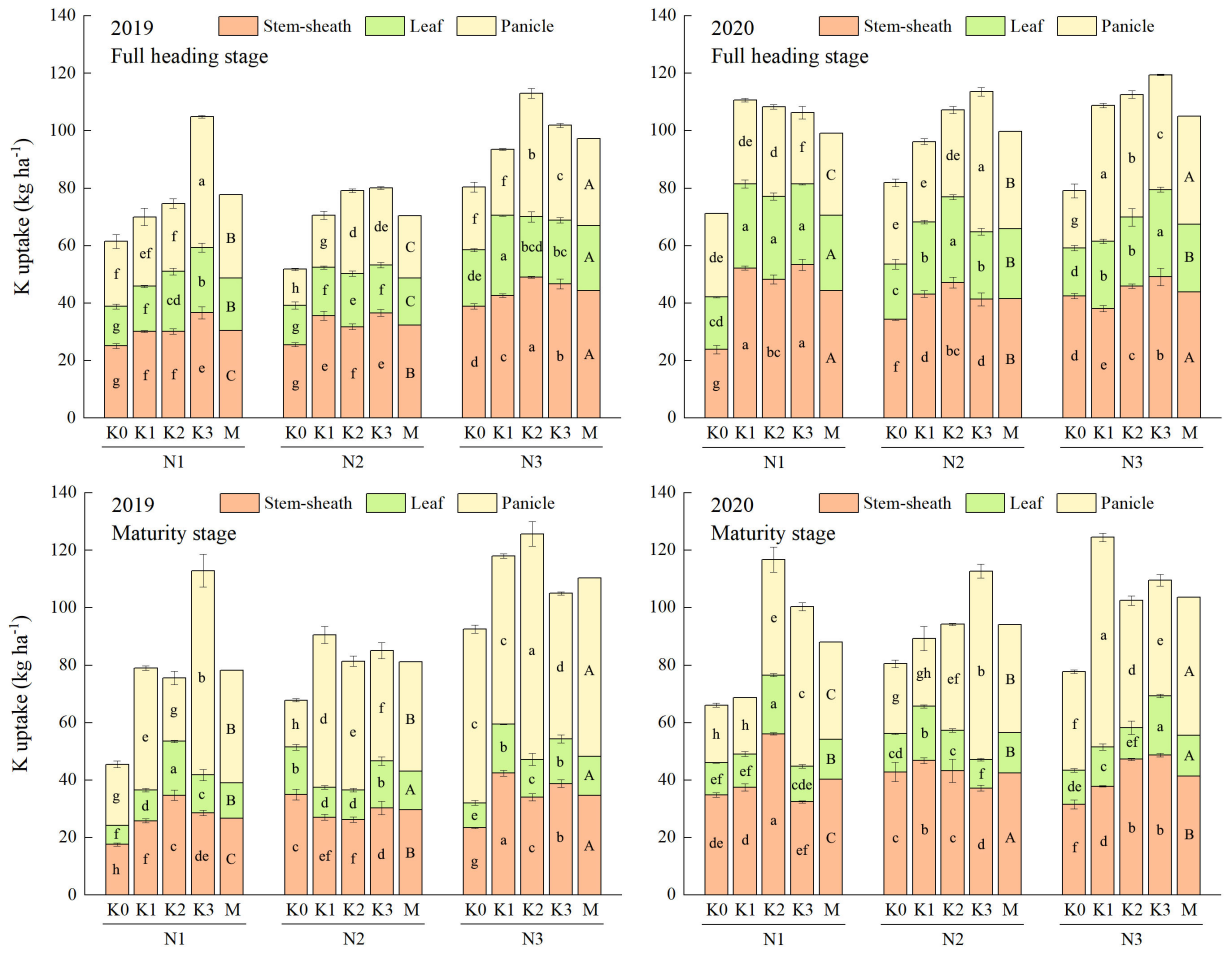
Effects of combined application of N and K on K uptake of YXY 2115 at full heading stage (above) and maturity stage (below). K0, K1, K2 and K3 refer to the different potassium fertilizer treatments (0, 90, 135 and 180 kg ha^-1^, respectively). N1, N2 and N3 refer to the different nitrogen fertilizer treatments (135, 180 and 225 kg ha^-1^, respectively). M represents the average of different potassium fertilizer levels under the same nitrogen fertilizer level. Different lowercase (uppercase) letters in the same color column mean the significant difference between treatments levels at *p* < 0.05. The data presented are the mean ± standard deviation, *n* = 3.

Increasing application of N could improve the uptake of both N and K (except K uptake in stem-sheath and leaf in 2020). The uptake of N and K could be further increased by applying appropriate K application, with a more obvious effect observed at high N level (**Figures 3**, **4**). Co-application of N and K had extremely significant interactive effects on the uptake of N and K in the aboveground organs of rice (**Supplementary Table S2**). The highest uptakes of N and K in stem-sheath, leaf and panicle were observed under N3 at both full heading stage and maturity stage.

At maturity stage, the maximum increases were obtained under N3K1 (48.20%) in 2019 and N3K2 (91.29%) in 2020 for the total uptake of N, and under N3K2 (176.30%) in 2019 and N3K1 (88.21%) in 2020 for the total uptake of K, respectively, when compared with other treatments.

### 3.3 Use efficiency of N and K

No obvious and consistent changes were observed in the response of N and K use efficiency indexes to the combined application of N and K in two years test results. NRE decreased but KHI increased with increasing N levels. Although the application of K could increase NRE, NAE, NCR and KCR within a certain range, it resulted in a decrease KAE (**Tables 2**, **3**). Variance analysis showed that co-application of N and K had extremely significant interactive effects only on recovery efficiency and harvest index of N and K.

**Table 2:** Effects of combined application of N and K on N use efficiency of YXY 2115.

Treatment		NRE (%)	2020	NAE (kg kg^-1^)	2020	NCR (%)	2020	NHI (%)	2020
Year		2019		2019		2019		2019	
N1	K0	14.32 ± 4.28f	17.97 ± 2.79de	0.39 ± 0.69d	0.79 ± 0.35d	0.79 ± 1.37d	1.63 ± 0.73e	55.97 ± 0.12a	48.66 ± 0.94ab
	K1	24.44 ± 1.70d	20.39 ± 2.57cde	1.55 ± 0.67abcd	1.66 ± 0.23ab	3.08 ± 1.30bcd	3.38 ± 0.46cd	54.22 ± 1.02ab	46.29 ± 0.48c
	K2	37.19 ± 1.40b	24.30 ± 3.83c	1.78 ± 1.23abc	1.84 ± 0.02a	3.52 ± 2.31abcd	3.73 ± 0.03bcd	50.79 ± 0.02c	47.42 ± 0.92bc
	K3	41.67 ± 1.21a	44.64 ± 1.50a	1.80 ± 1.20abc	1.91 ± 0.38a	3.56 ± 2.25abcd	3.86 ± 0.74bc	47.74 ± 0.35d	50.34 ± 1.41a
	Mean	29.41A	26.83A	1.38B	1.55A	2.74B	3.15B	52.18A	48.18A
N2	K0	12.57 ± 2.58f	16.33 ± 1.91e	0.55 ± 0.55cd	1.18 ± 0.03c	1.47 ± 1.43cd	3.19 ± 0.09d	55.95 ± 0.27a	41.85 ± 1.11d
	K1	24.34 ± 0.23d	19.94 ± 2.17cde	1.75 ± 0.65abc	1.73 ± 0.04a	4.56 ± 1.63ab	4.61 ± 0.10a	52.62 ± 1.17bc	47.69 ± 1.16bc
	K2	29.27 ± 2.43c	22.42 ± 3.85cd	2.36 ± 0.44a	1.78 ± 0.05a	6.05 ± 1.07a	4.74 ± 0.13a	44.70 ± 2.10e	47.16 ± 1.61bc
	K3	35.54 ± 0.61b	40.56 ± 3.53a	1.90 ± 0.13ab	1.60 ± 0.02ab	5.01 ± 0.33ab	4.28 ± 0.04ab	47.70 ± 2.64d	40.22 ± 1.70de
	Mean	25.43B	24.81AB	1.64A	1.57A	4.27A	4.21A	50.24B	44.23B
N3	K0	12.37 ± 1.27f	15.22 ± 2.06e	0.70 ± 0.14bcd	1.10 ± 0.03c	2.33 ± 0.46bcd	3.70 ± 0.09bcd	53.62 ± 2.54ab	41.96 ± 1.41d
	K1	20.68 ± 2.22e	18.27 ± 2.06de	1.25 ± 0.72abcd	1.39 ± 0.00bc	4.10 ± 2.32abc	4.64 ± 0.01a	56.33 ± 0.42a	40.13 ± 0.29de
	K2	24.04 ± 1.20de	20.27 ± 2.62cde	1.46 ± 0.07abcd	1.40 ± 0.02bc	4.74 ± 0.20ab	4.68 ± 0.04a	53.02 ± 1.64bc	38.29 ± 0.07e
	K3	30.06 ± 0.13c	34.09 ± 3.41b	1.59 ± 0.12abcd	1.27 ± 0.04c	5.14 ± 0.35ab	4.27 ± 0.12ab	47.16 ± 0.67de	50.34 ± 0.73a
	Mean	21.79C	21.96B	1.25B	1.29B	4.08A	4.32A	52.53A	42.68B
N		45.79**	9.16**	1.15	10.41**	2.33	44.58**	9.22**	78.98**
K		226.11**	125.14**	5.81**	29.74**	7.29**	40.42**	62.96**	12.71**
N × K		6.65**	1.49	0.34	4.92	0.75	3.48*	9.03**	49.50**
Year		8.04**		0.70		1.06		44.23**	

N1, N2 and N3 refer to the different nitrogen fertilizer treatments (135, 180 and 225 kg ha^-1^, respectively). K0, K1, K2 and K3 refer to the different potassium fertilizer treatments (0, 90, 135 and 180 kg ha^-1^, respectively). NRE, NAE, NCR and NHI represent nitrogen recovery efficiency, nitrogen agronomic efficiency, nitrogen contribution rate and nitrogen harvest index, respectively. Different lowercase letters followed the values in the same column mean the significant difference of the different combined application of N and K levels at p < 0.05. Different uppercase letters mean the significant difference of different average N levels at p < 0.05. ANOVA p values and symbols were defined as: * p < 0.05; ** p < 0.01; ns: p > 0.05. The data presented are the mean ± standard deviation, n = 3.

**Table 3:** Effects of combined application of N and K on K use efficiency of YXY 2115.

Treatment		KRE (%)	2020	KAE (kg kg^-1^)	2020	KCR (%)	2020	KHI (%)	2020
Year		2019		2019		2019		2019	
N1	K0	–	–	–	–	–	–	46.77 ± 1.84fg	30.39 ± 0.47f
	K1	37.27 ± 1.57a	2.94 ± 2.02e	1.75 ± 2.03b	1.31 ± 0.22a	2.32 ± 2.68d	1.78 ± 0.29ab	53.80 ± 0.47d	28.75 ± 0.14fg
	K2	22.25 ± 3.51c	37.47 ± 2.49b	1.39 ± 0.59c	1.06 ± 0.35ab	2.76 ± 1.08c	2.14 ± 0.70a	29.22 ± 1.58h	34.39 ± 2.22e
	K3	37.46 ± 4.09a	19.04 ± 0.48c	1.06 ± 0.41d	0.84 ± 0.55bc	2.32 ± 1.77d	2.26 ± 1.46a	63.01 ± 1.38b	55.34 ± 1.08b
	Mean	32.33A	19.82B	1.40B	1.07A	2.47B	2.06A	48.20B	37.22B
N2	K0	–	–	–	–	–	–	24.12 ± 0.64i	30.24 ± 2.39f
	K1	25.20 ± 1.64bc	9.76 ± 2.78d	2.40 ± 2.14a	1.09 ± 0.12ab	3.13 ± 2.77b	1.46 ± 0.15abc	58.61 ± 0.47c	26.43 ± 3.60g
	K2	10.01 ± 1.53d	10.19 ± 5.45d	2.42 ± 0.38a	0.80 ± 0.11bc	4.65 ± 0.73a	1.60 ± 0.21ab	55.14 ± 1.29d	39.13 ± 1.56d
	K3	8.33 ± 2.51d	17.92 ± 0.61c	0.80 ± 0.46e	0.42 ± 0.05de	2.11 ± 1.23e	1.12 ± 0.12bc	45.20 ± 0.18g	58.19 ± 0.37ab
	Mean	14.51B	12.62C	1.87A	0.77AB	3.30A	1.40AB	45.77C	38.50B
N3	K0	–	–	–	–	–	–	65.38 ± 0.38a	44.18 ± 0.98c
	K1	28.26 ± 3.51b	51.88 ± 1.94a	1.38 ± 2.15c	0.73 ± 0.08bcd	1.81 ± 0.62f	0.98 ± 0.10bc	49.57 ± 0.73e	58.65 ± 0.90a
	K2	24.52 ± 7.36bc	18.32 ± 0.74c	1.22 ± 0.27c	0.51 ± 0.03cde	2.46 ± 0.66d	1.02 ± 0.04bc	62.45 ± 0.45b	43.21 ± 1.77c
	K3	6.90 ± 2.49d	17.66 ± 2.08c	1.11 ± 0.15d	0.22 ± 0.02e	2.88 ± 0.40c	0.60 ± 0.04c	48.34 ± 1.52ef	36.74 ± 0.84de
	Mean	19.89B	29.28A	1.24C	0.49B	2.38B	0.87B	56.44A	45.70A
N		58.60**	99.33**	0.44	13.69**	0.93	10.29**	333.11**	90.74**
K		34.00**	6.06**	2.44	12.42**	0.90	0.51	113.81**	143.3**
N × K		17.22**	163.53**	0.50	0.18	0.76	0.60	637.43**	166.00**
Year		54.18**		0.79		0.96		168.00**	

N1, N2 and N3 refer to the different nitrogen fertilizer treatments (135, 180 and 225 kg ha^-1^, respectively). K0, K1, K2 and K3 refer to the different potassium fertilizer treatments (0, 90, 135 and 180 kg ha^-1^, respectively). KRE, KAE, KCR and KHI represent potassium recovery efficiency, potassium agronomic efficiency, potassium contribution rate and potassium harvest index, respectively. Different lowercase letters followed the values in the same column mean the significant difference of the different combined application of N and K levels at p < 0.05. Different uppercase letters mean the significant difference of different average N levels at p < 0.05. ANOVA p values and symbols were defined as: * p < 0.05; ** p < 0.01; ns: p > 0.05. The data presented are the mean ± standard deviation, n = 3.

Compared with other treatments, the maximum increases were obtained under N1K3 (193.30-236.86%) in both years for NRE, and under N1K3 (442.90%) in 2019 and N3K1 (1664.63%) in 2020 for KRE. And the maximum increases were obtained under N3K1 (19.44%) in 2019 and N3K3 (31.47%) in 2020 for NHI, and under N3K0 (171.06%) in 2019 and N3K1 (121.91%) in 2020 for KHI.

### 3.4 Expression of N and K metabolism-related genes

**Figure 5:**
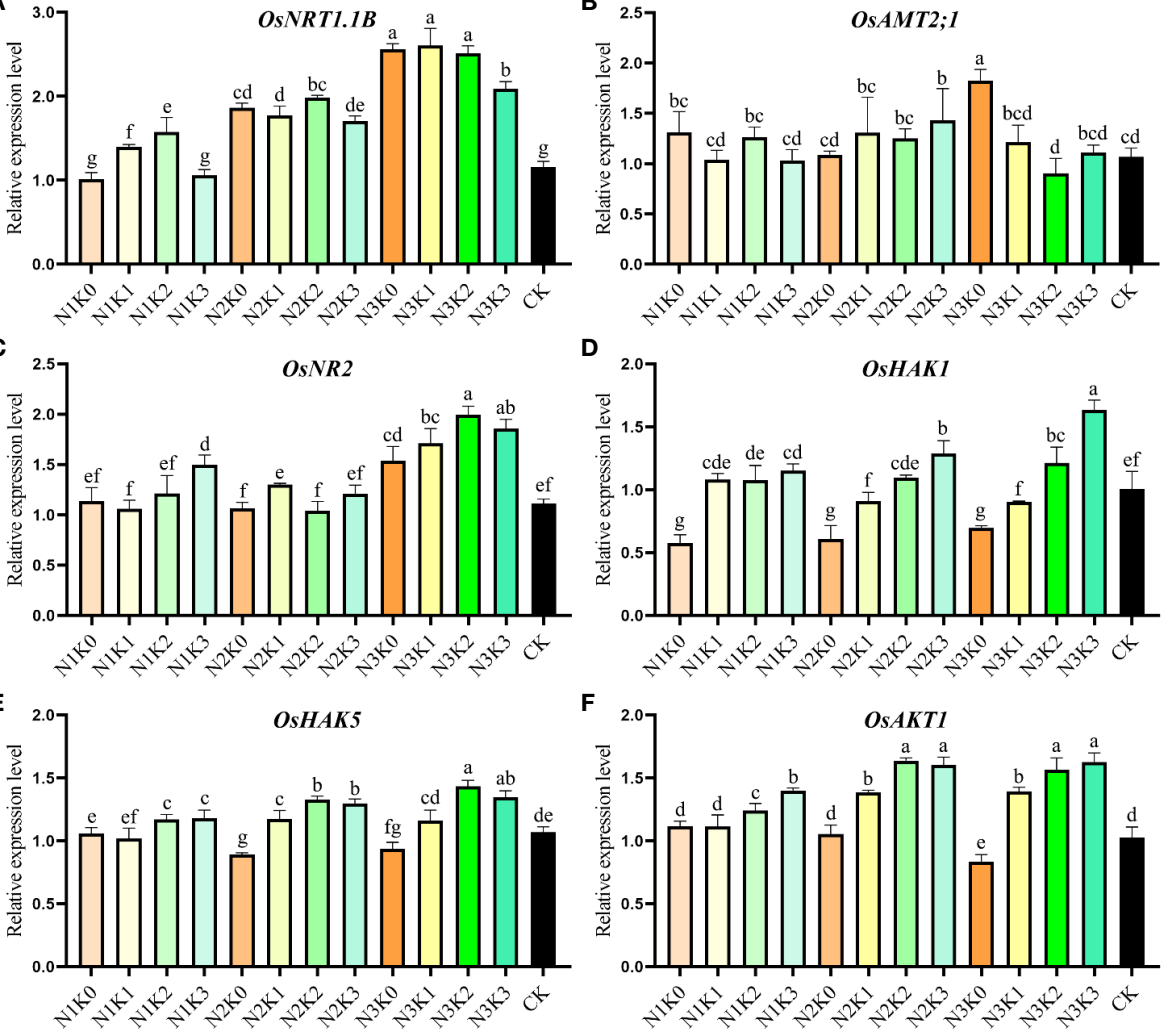
Effects of combined application of N and K on expression levels of N and K metabolism-related genes in rice grains at mid-filling stage. N1, N2 and N3 refer to the different nitrogen fertilizer treatments (135, 180 and 225 kg ha^-1^, respectively). K0, K1, K2 and K3 refer to the different potassium fertilizer treatments (0, 90, 135 and 180 kg ha^-1^, respectively). Different lowercase letters mean the significant difference between treatments levels at *p* < 0.05. The data presented are the mean ± standard deviation, *n* = 3.

Six genes were selected for analysis in response to the different fertilizer application rates in hybrid rice cultivar YXY 2115, considering their significant roles in N and K metabolism. The results showed that *OsNRT1.1B*, *OsNR2*, *OsHAK1*, *OsHAK5* and *OsAKT1* had high expression level under high application rates of both N and K except *OsAMT2;1*, which were consistent with the above results of N and K uptake (**Figure 5**). The peak expression levels of *OsNRT1.1B* and *OsAMT2;1* were obtained under N3K1 and N3K0, respectively. *OsNR2* and *OsHAK5* got their highest expression levels under N3K2. Meanwhile, *OsHAK1* and *OsAKT1* achieved the highest expression levels under N3K3.

### 3.5 Yield and yield components

With increasing N levels, grain yield (GY), panicle number (PN) and spikelet per panicle (SP) increased, while seed setting rate (SR) showed the opposite trend. No significant difference was found in 1000-grain weight (GW) among different treatments. The combination of appropriate N levels with a high K level could further improve yield and yield components (**Table 4**). Variance analysis showed that co-application of N and K had extremely significant interactive effects on SP. The highest GY, PN and SP were observed under N3. Compared with other treatments, the maximum increases of GY and SR were obtained under N2K2 (3.27-5.61%) and N1K3 (16.32-20.53%), respectively. And the maximum increases were obtained under N2K3 (117.97%) in 2019 and N3K3 (39.06%) in 2020 for PN, and under N3K2 (29.10%) in 2019 and N2K3 (30.78%) in 2020 for SP.

**Table 4:** Effects of combined application of N and K on yield and yield components of YXY 2115.

Treatment		PN (×10^4^ ha^-1^)	2020	SP	2020	GW (g)	2020	SR (%)	2020	GY (kg ha^-1^)	2020
Year		2019		2019		2019		2019		2019	
N1	K0	191.70 ± 0.36j	182.88 ± 2.05f	144.49 ± 1.11e	133.97 ± 5.46c	32.15 ± 0.96a	31.47 ± 0.39b	83.45 ± 0.49de	82.48 ± 0.13cdef	6638.06 ± 92.51d	6531.56 ± 47.64e
	K1	194.55 ± 0.28i	188.59 ± 8.08ef	151.13 ± 1.79d	145.01 ± 5.48c	32.20 ± 0.45a	32.67 ± 0.85ab	85.18 ± 0.26d	84.23 ± 2.97bcd	6795.48 ± 90.64bcd	6649.71 ± 31.68cd
	K2	204.55 ± 0.65h	202.88 ± 4.01d	169.81 ± 4.07c	158.75 ± 2.65b	32.33 ± 2.09a	33.15 ± 0.41a	89.87 ± 0.36b	86.30 ± 1.86b	6826.22 ± 165.97abcd	6674.25 ± 2.15bcd
	K3	210.89 ± 1.68f	214.31 ± 10.93c	170.13 ± 1.66c	161.1 ± 20.98b	32.36 ± 1.74a	33.49 ± 0.06a	92.63 ± 0.10a	92.59 ± 2.16a	6828.74 ± 161.61abcd	6683.02 ± 51.52bc
	Mean	200.42C	197.17C	158.89C	149.71B	32.26A	32.70A	87.78A	86.40A	6772.13A	6634.64B
N2	K0	207.68 ± 1.28g	194.31 ± 2.27def	150.32 ± 0.91d	134.97 ± 8.40c	31.98 ± 1.25a	31.41 ± 0.21b	79.56 ± 2.47f	80.92 ± 0.55def	6684.24 ± 98.02cd	6637.02 ± 5.86d
	K1	213.89 ± 1.46e	200.02 ± 12.22de	173.08 ± 2.31bc	168.16 ± 2.32b	32.88 ± 0.37a	32.22 ± 1.71ab	82.97 ± 0.44e	81.93 ± 2.09cdef	6900.52 ± 116.44ab	6735.45 ± 6.70a
	K2	224.87 ± 1.36ab	225.74 ± 6.01c	183.30 ± 2.15a	172.31 ± 1.21ab	33.13 ± 1.30a	32.39 ± 1.15ab	83.66 ± 0.47de	83.79 ± 4.15bcde	7010.27 ± 80.22a	6745.02 ± 9.56a
	K3	226.15 ± 0.90a	237.17 ± 12.07b	185.27 ± 1.29a	175.20 ± 3.60a	33.14 ± 1.03a	32.50 ± 0.48ab	87.48 ± 1.03c	85.49 ± 3.79bc	6828.29 ± 23.92abcd	6712.45 ± 2.64ab
	Mean	218.15B	214.31B	172.99B	162.66A	32.78A	32.13A	83.42B	83.03B	6855.83A	6707.49A
N3	K0	218.11 ± 0.49d	225.74 ± 11.92c	174.72 ± 2.41b	161.90 ± 1.39b	31.25 ± 0.77a	31.36 ± 0.24b	76.85 ± 1.98g	79.60 ± 1.92f	6743.24 ± 31.70bcd	6672.03 ± 6.15bcd
	K1	222.96 ± 0.54c	237.17 ± 10.12b	185.26 ± 1.12a	161.21 ± 4.02b	32.54 ± 1.50a	31.69 ± 0.22b	76.88 ± 0.52g	79.94 ± 0.35ef	6867.69 ± 161.96abc	6738.08 ± 0.71a
	K2	223.17 ± 0.71c	245.74 ± 8.83ab	186.54 ± 1.05a	169.81 ± 2.95ab	32.60 ± 0.72a	32.11 ± 0.97ab	80.16 ± 1.44f	81.05 ± 1.02def	6913.54 ± 14.86ab	6740.83 ± 3.16a
	K3	223.87 ± 1.24bc	254.31 ± 16.37a	186.24 ± 0.91a	159.69 ± 2.15b	32.68 ± 0.93a	32.16 ± 0.58ab	82.06 ± 2.35e	81.14 ± 0.92def	6942.92 ± 25.96ab	6712.04 ± 8.81ab
	Mean	222.03A	240.74A	183.19A	163.15A	32.27A	31.83A	78.99C	80.43B	6866.85A	6715.75A
N		1528.06**	61.32**	474.44**	13.31**	0.75	3.99*	140.44**	21.87**	3.00	46.06**
K		375.92**	22.93**	302.30**	19.46**	1.20	5.20**	58.32**	10.21**	8.24**	41.35**
N × K		49.61**	0.77	32.17**	5.44**	0.21	0.41	2.571*	2.28	0.72	3.40*
Year		6.11**		2.31*		0.68		1.66		0.99	

N1, N2 and N3 refer to the different nitrogen fertilizer treatments (135, 180 and 225 kg ha^-1^, respectively). K0, K1, K2 and K3 refer to the different potassium fertilizer treatments (0, 90, 135 and 180 kg ha^-1^, respectively). PN, SP, GW, SR and GY represent panicle number, spikelet number per panicle, 1000-grain weight, seed setting rate and grain yield, respectively. Different lowercase letters followed the values in the same column mean the significant difference of the different combined application of N and K levels at p < 0.05. Different uppercase letters mean the significant difference of different average N levels at p < 0.05. ANOVA p values and symbols were defined as: * p < 0.05; ** p < 0.01; ns: p > 0.05. The data presented are the mean ± standard deviation, n = 3.

Regression analysis of N and K application rates and yield was carried out. By establishing a binary quadratic equation, the fertilizer response function equation between N and K application rates and yield was obtained.


$${\text{Y}}_{2019}=-0.0179{\text{N}}^{2}+7.4833\text{N}-0.0112{\text{K}}^{2}+3.0036\text{K}+0.0003\text{NK}+5944.5694$$

$${\text{Y}}_{2020}=-0.0159{\text{N}}^{2}+7.3209\text{N}-0.0062{\text{K}}^{2}+2.8293\text{K}-0.0067\text{NK}+5833.8396$$

Here: Y represents the yield (kg ha^-1^), N represents the nitrogen application rate (kg ha^-1^), K represents the potassium application rate (kg ha^-1^).

**Figure 6:**
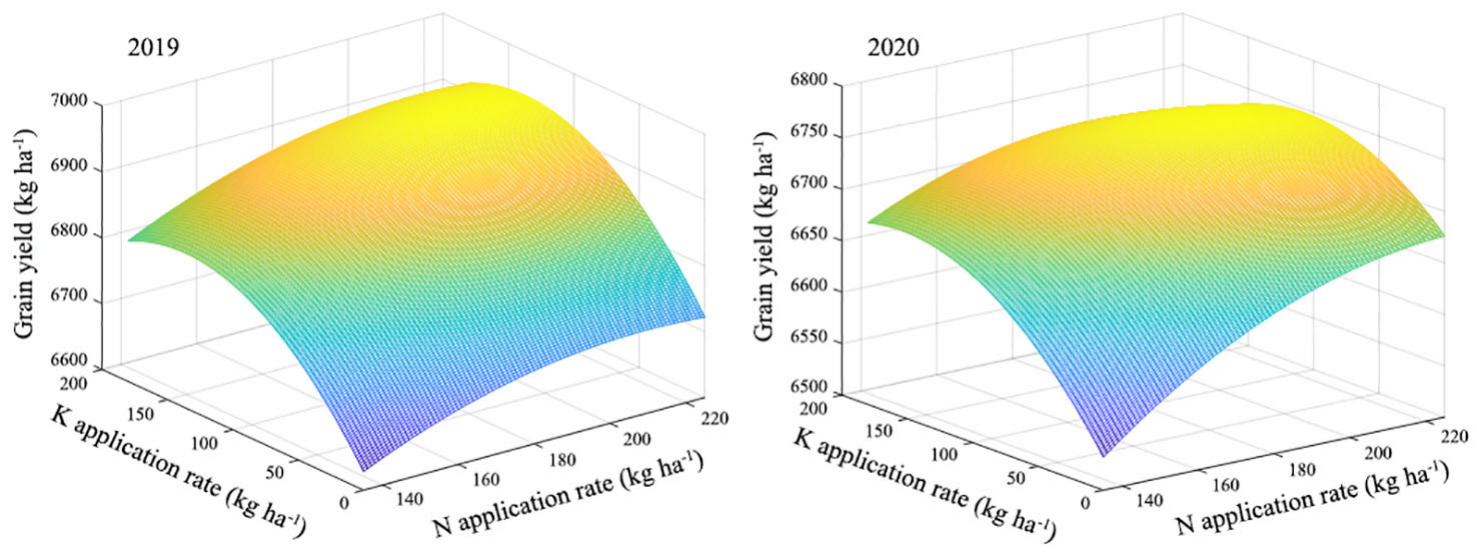
The fertilizer response function equation of relationship between rice yield and combined application of N and K. N and K represent nitrogen and potassium, respectively.

The results showed that the relationship between N and K application rates and yield could be described by binary quadratic equations, with each index exhibiting a maximum value (**Figure 6**). Considering both economic benefits and yield, in conjunction with the fertilizer response function equation, the optimal yield reached 6936.58 kg ha^-1^ in 2019. The recommended application rates for N and K were 210.18 kg ha^-1^ and 136.90 kg ha^-1^, respectively. In 2020, the highest yield achieved was 6750.54 kg ha^-1^ in 2020, with the suggested application rates of N and K being 205.98 kg ha^-1^ and 115.02 kg ha^-1^, respectively.

### 3.6 Rice quality

The application of N could improve rice appearance quality, but decreased milling quality, as well as cooking and eating quality. Conversely, the application of K had the opposite effect. Co-application of N and K had extremely significant interactive effects on various rice quality indexes, including milled rice rate (MR), head rice rate (HR), chalkiness rate (CR), chalkiness degree (CD), peak viscosity (PV), breakdown viscosity (BV), setback viscosity (SV) and taste value (TV) (**Tables 5**, **6**). The complete data for rice cooking and eating quality is available in **Supplementary Tables S1**, **S2**. However, there were no obvious changes in the response of RVA profile characters to the combined application of N and K. The highest MR, HR and TV were observed under N1, while the lowest CR and CD were observed under N1. The optimal rice milling quality and cooking and eating quality were obtained under N1K2, while the best rice appearance quality was obtained under N3K0. Compared with other treatments, the maximum increases in MR, HR, TV, CR and CD were 10.67-13.11%, 15.86-36.93%, 11.25-12.24%, 36.84-38.02% and 47.06-47.46%, respectively.

**Table 5:** Effects of combined application of N and K on milling and appearance quality of YXY 2115.

Treatment		BR (%)	2020	MR (%)	2020	HR (%)	2020	CR (%)	2020	CD (%)	2020
Year		2019		2019		2019		2019		2019	
N1	K0	74.19 ± 0.89cd	76.71 ± 0.41b	63.68 ± 0.87cd	64.33 ± 1.66cd	60.57 ± 0.39cd	46.63 ± 1.11d	8.34 ± 0.09e	7.80 ± 0.17f	2.26 ± 0.04e	2.00 ± 0.09ef
	K1	75.08 ± 0.66bc	77.03 ± 0.42ab	64.42 ± 0.06bc	65.71 ± 0.66bc	62.13 ± 1.12bc	51.36 ± 1.15c	10.34 ± 0.11c	11.40 ± 1.24ab	2.60 ± 0.05d	2.70 ± 0.20cd
	K2	77.93 ± 0.08a	77.41 ± 0.75ab	67.73 ± 0.74a	71.27 ± 0.80a	65.75 ± 0.46a	63.85 ± 0.50a	10.86 ± 0.07b	12.00 ± 0.62a	2.80 ± 0.06c	2.80 ± 0.28bc
	K3	73.42 ± 1.68de	75.95 ± 4.64b	64.19 ± 0.47c	65.13 ± 0.26bc	61.69 ± 1.29bc	50.35 ± 0.14c	11.40 ± 0.08a	12.10 ± 0.25a	2.94 ± 0.06bc	3.10 ± 0.12b
	Mean	75.16A	76.78A	65.01A	66.61A	62.54A	53.05A	10.24A	10.83A	2.65A	2.65A
N2	K0	72.91 ± 0.21ef	74.89 ± 0.65b	62.77 ± 0.41de	66.24 ± 0.99bc	59.69 ± 2.35de	52.07 ± 1.50c	7.54 ± 0.25f	9.30 ± 0.96e	1.86 ± 0.12f	2.10 ± 0.14e
	K1	75.40 ± 0.06b	79.59 ± 1.09a	65.33 ± 0.40b	67.00 ± 0.66b	62.59 ± 0.69b	53.93 ± 0.34b	9.66 ± 0.31d	9.90 ± 0.69de	2.06 ± 0.26ef	2.40 ± 0.06d
	K2	72.96 ± 0.15ef	75.92 ± 1.62b	61.92 ± 0.46ef	65.19 ± 2.21bc	58.73 ± 1.05de	51.65 ± 0.96c	9.80 ± 0.33d	10.30 ± 1.04cd	3.14 ± 0.05b	2.50 ± 0.19d
	K3	72.91 ± 0.22ef	75.00 ± 0.53b	59.62 ± 0.68g	64.47 ± 0.71cd	58.04 ± 0.96ef	51.85 ± 1.72c	11.14 ± 0.26ab	11.00 ± 0.26bc	3.54 ± 0.17a	2.90 ± 0.32bc
	Mean	73.55B	76.35AB	62.41B	65.73A	59.76B	52.38A	9.54B	10.13B	2.65A	2.48B
N3	K0	72.48 ± 0.21ef	74.86 ± 0.89b	61.21 ± 0.17f	64.67 ± 0.15cd	58.94 ± 0.11de	48.19 ± 0.39d	7.20 ± 0.12f	7.50 ± 0.20f	1.86 ± 0.02f	1.80 ± 0.17f
	K1	73.45 ± 0.90de	76.29 ± 1.85ab	62.22 ± 0.87ef	64.67 ± 0.49cd	59.69 ± 0.74de	48.26 ± 0.06d	7.54 ± 0.16f	9.20 ± 0.15e	1.94 ± 0.12f	2.50 ± 0.05d
	K2	74.42 ± 0.14bcd	77.11 ± 0.27ab	64.30 ± 0.27bc	65.14 ± 0.26bc	62.75 ± 0.97b	51.89 ± 0.63c	8.66 ± 0.24e	9.80 ± 0.14de	2.26 ± 0.13e	3.10 ± 0.02b
	K3	71.85 ± 0.18f	74.73 ± 0.81b	61.20 ± 0.99f	63.01 ± 2.70d	56.75 ± 1.34f	48.26 ± 0.42d	10.46 ± 0.21c	10.80 ± 0.61bc	3.06 ± 0.10b	3.40 ± 0.07a
	Mean	73.05B	75.75B	62.23B	64.37B	59.53B	49.15B	8.47C	9.33C	2.28B	2.70A
N		34.32**	1.21	79.68**	9.99**	27.55**	63.62**	226.63**	16.20**	39.32**	6.00**
K		27.45**	4.37*	45.36**	9.47**	19.55**	98.23**	403.55**	38.91**	185.43**	78.39**
N × K		9.93**	1.54	23.15**	7.16**	8.96**	61.62**	17.66**	4.22**	20.98**	5.16**
Year		1.16		2.90**		14.69**		3.17**		14.04**	

N1, N2 and N3 refer to the different nitrogen fertilizer treatments (135, 180 and 225 kg ha^-1^, respectively). K0, K1, K2 and K3 refer to the different potassium fertilizer treatments (0, 90, 135 and 180 kg ha^-1^, respectively). BR, MR, HR, CR and CD represent brown rice rate, milled rice rate, head rice rate, chalkiness rate and chalkiness degree, respectively. Different lowercase letters followed the values in the same column mean the significant difference of the different combined application of N and K levels at p < 0.05. Different uppercase letters mean the significant difference of different average N levels at p < 0.05. ANOVA p values and symbols were defined as: * p < 0.05; ** p < 0.01; ns: p > 0.05. The data presented are the mean ± standard deviation, n = 3.

**Table 6:** Effects of combined application of N and K on cooking and eating quality of YXY 2115. .

Treatment		PV (RVU)	2020	BV (RVU)	2020	SV (RVU)	2020	TV	2020
Year		2019		2019		2019		2019	
N1	K0	248.46 ± 3.80ef	158.83 ± 4.38g	116.33 ± 1.36bc	60.58 ± 4.23g	-25.54 ± 0.27e	20.83 ± 7.78a	86.00 ± 0.00bc	80.00 ± 1.73f
	K1	261.67 ± 6.50b	240.92 ± 0.60b	121.96 ± 7.78ab	101.33 ± 0.67ab	-23.71 ± 2.15de	-19.25 ± 3.09de	86.33 ± 0.58b	87.33 ± 1.53abc
	K2	255.38 ± 2.30bcd	255.75 ± 8.31a	124.67 ± 1.99a	106.33 ± 3.05a	-32.63 ± 1.96f	-24.42 ± 3.43e	88.67 ± 0.58a	89.00 ± 0.00a
	K3	251.71 ± 2.96cde	228.33 ± 4.90c	108.96 ± 4.47de	92.75 ± 2.78cd	-14.29 ± 3.53b	-12.25 ± 6.15cd	86.33 ± 0.58b	88.33 ± 1.53ab
	Mean	254.31A	220.96A	117.98A	90.25B	-24.04B	-8.77AB	86.83A	86.17A
N2	K0	257.46 ± 1.55bc	221.08 ± 0.77de	126.75 ± 0.91a	94.58 ± 2.26c	-33.29 ± 4.01f	-13.42 ± 2.29cd	83.00 ± 1.00d	83.33 ± 1.15e
	K1	270.17 ± 3.68a	226.25 ± 2.56cd	123.92 ± 2.42a	97.75 ± 3.49bc	-28.04 ± 2.00ef	-12.08 ± 3.99cd	84.33 ± 1.15cd	84.33 ± 0.58de
	K2	242.46 ± 1.79fg	235.33 ± 2.72b	112.00 ± 1.66cd	104.67 ± 2.11a	-17.96 ± 2.72bc	-18.92 ± 0.93de	86.00 ± 1.00bc	86.00 ± 1.00cd
	K3	232.96 ± 4.93h	215.25 ± 1.13e	107.25 ± 3.19de	88.17 ± 0.94de	-14.42 ± 3.37b	-2.67 ± 4.24b	85.67 ± 1.53bc	85.67 ± 0.58cd
	Mean	250.76B	224.48A	117.48A	96.29A	-23.43B	-11.77B	84.75B	84.83B
N3	K0	249.67 ± 1.38de	202.83 ± 2.64f	113.08 ± 2.30cd	79.42 ± 2.23f	-20.08 ± 3.36cd	-0.67 ± 1.20b	83.00 ± 1.00d	80.67 ± 0.58f
	K1	252.38 ± 0.07cde	221.75 ± 1.67cde	121.29 ± 0.16ab	94.17 ± 2.79c	-31.67 ± 2.74f	-12.25 ± 3.70cd	84.33 ± 0.58cd	87.00 ± 1.00bc
	K2	239.13 ± 2.67g	226.08 ± 2.01cd	103.58 ± 0.75e	86.42 ± 2.68e	-6.00 ± 2.63a	-6.50 ± 2.71bc	82.67 ± 0.58d	84.33 ± 0.58de
	K3	232.46 ± 4.66h	206.75 ± 2.85f	105.88 ± 2.88e	79.67 ± 4.88f	-13.00 ± 4.07b	0.25 ± 2.51b	79.00 ± 2.65e	83.67 ± 0.58e
	Mean	243.41C	214.35B	110.96B	84.92C	-17.69A	-4.79A	82.25C	83.92B
N		30.69**	25.32**	18.41**	45.84**	17.31**	9.36**	38.52**	16.29**
K		67.43**	270.59**	38.49**	103.32**	44.90**	44.10**	4.40*	82.83**
N × K		14.37**	109.19**	10.65**	40.06**	22.25**	25.12**	6.91**	16.78**
Year		67.66**		24.03**		20.80**		10.38**	

N1, N2 and N3 refer to the different nitrogen fertilizer treatments (135, 180 and 225 kg ha^-1^, respectively). K0, K1, K2 and K3 refer to the different potassium fertilizer treatments (0, 90, 135 and 180 kg ha^-1^, respectively). PV, BV, SV and TV represent peak viscosity, breakdown viscosity, setback viscosity and taste value, respectively. Different lowercase letters followed the values in the same column mean the significant difference of the different combined application of N and K levels at p < 0.05. Different uppercase letters mean the significant difference of different average N levels at p < 0.05. ANOVA p values and symbols were defined as: * p < 0.05; ** p < 0.01; ns: p > 0.05. The data presented are the mean ± standard deviation, n = 3.

## 4 Discussion

### 4.1 Nutrient uptake and use efficiency

**Figure 7:**
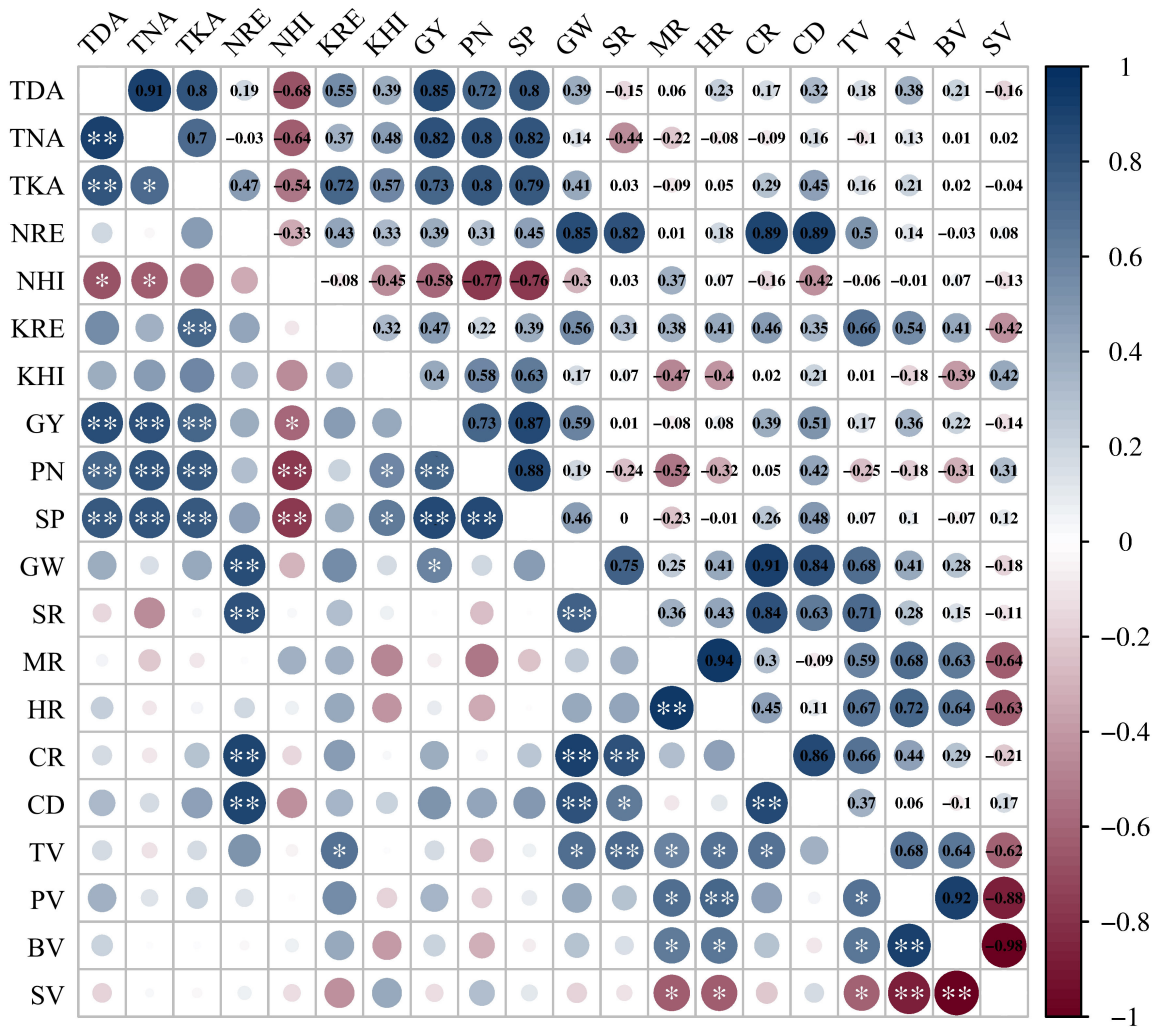
Correlation coefficients of nutrients absorption and utilization characteristics with rice yield, yield components and rice quality. TDA, TNA, TKA, NRE, NHI, KRE, KHI, GY, PN,SP, GW, SR, MR, HR, CR, CD, TV, PV, BV and SV represent total dry matter accumulation at maturity stage, total nitrogen accumulation at maturity stage, total potassium accumulation at maturity stage, nitrogen recovery efficiency, nitrogen harvest index, potassium recovery efficiency, potassium harvest index, grain yield, panicle number, spikelet number per panicle, 1000-grain weight, seed setting rate, milled rice rate, head rice rate, chalkiness rate, chalkiness degree, taste value, peak viscosity, breakdown viscosity and setback viscosity, respectively. ANOVA *p* values and symbols were defined as: * *p* < 0.05; ** *p* < 0.01; ns: *p* > 0.05.

The growth of rice and the formation of rice yield are closely linked to nutrient uptake. Studies have reported that the accumulation of DM increased with increasing N levels (Sun, 2019), and the N accumulation of rice all increased at each growth stage (Tang et al., 2019). The combined application of N and K could significantly alleviate the limitations to photosynthesis, especially the biological constraints (Hou et al., 2018). In the present study, the DM accumulation of each vegetative organ in the aboveground part of YXY 2115 increased with increasing N levels at both full heading stage and maturity stage. Notably, the uptakes of N and K in stem-sheaths and leaves decreased, while showing a significantly increase in panicles, in agreement with the previous studies (Li et al., 2014; Yan, 2018; Jiang et al., 2020). Compared with N1, the total DM accumulation at maturity stage increased by 14.79-18.64%%. As more N was gradually applied, the uptakes of N and K in stem-sheaths, leaves and panicles all increased. Compared with N1, the total uptake of N and K under N3 at maturity stage increased by 5.89-24.94%% and 17.68-40.99%, respectively. The enhancement of N and K uptake could be further optimized through the appropriate application of K, with a more pronounced effect observed under high N levels. However, the DM accumulation showed a trend of first increasing and then decreasing with increasing K under moderate and higher N levels, indicating that while appropriate application of K under high N levels increased DM accumulation, excessively high N and K levels may hinder this process. This phenomenon might be attributed to the decrease in leaf Rubisco enzyme activity under high N levels, subsequently limiting the photosynthetic N use efficiency (Li, 2011). Correlation analysis is statistical method that is used to discover if there is a relationship between two variables, and how strong that relationship may be. Hence, correlation analysis was conducted on indexes that were relatively important in terms of rice nutrient absorption and utilization, yield, and quality, as well as those exhibiting significant interactive effects when N and K were applied together. Correlation analysis showed that GY was significantly positively correlated with TDA, TNA and TKA at maturity stage (**Figure 7**). These results indicated that rational co-application of N (225 kg ha^-1^) and K (90-135 kg ha^-1^) could promote the N and K uptake and the N and K accumulation in the aboveground parts, thereby maintaining or increasing yield. As DM accumulation forms the foundation for rice yield. Taken together, high N levels (180-225 kg ha^-1^) combined with appropriate application of K (135 kg ha^-1^) could not only mitigate fertilizer waste but also facilitate the establishment of a high-yield population in rice production.

Fertilizer use efficiency is an important indicator for assessing the transportation and distribution of nutrients in plant organs (Zhou et al., 2020). The application rate of N has a significant effect on NRE, NAE and NHI. As N levels increased, N accumulation in rice increased significantly. However, the proportion of N in panicles decreased while its proportion in stem-sheaths increased, resulting in a decrease in NRE, NAE and NHI (Dong et al., 2015; Cheng et al., 2018). K has been observed to enhance the activities of nitrate reductase and aminotransferases, indicating a positive role of K in N metabolism (Ahanger et al., 2015; Hu et al., 2016). In our study, as more N was gradually applied, NRE decreased and KHI increased. Simultaneously, NRE, NAE, NCR and KCR increased, while KAE decreased with increasing K at the same N level. The observed changes in nutrients use efficiency and yield aligned with the principle of diminishing returns for fertilizer (Lu, 2003). Most researchers addressed that the application of N within a certain range could lead to a decrease in NRE, which was consistent with our results. However, variations in other indexes in the two-year field experiments were either inconsistent or not conspicuous, which differed with the results of previous studies. This might be related to the unique characteristics of the YXY 2115 cultivar. The varying sensitivity of different rice cultivars to N and K could potentially be a key factor affecting fertilizer absorption and utilization (Qiu et al., 2016), necessitating further research for a comprehensive understanding.

### 4.2 Expression of N and K metabolism-related genes

There are two main pathways through which plants acquire nutrients: direct nutrient absorption, where plant roots directly assimilate nutrients via ion channels or transporters from the soil; and indirect nutrient absorption, through which plants obtain nutrients from the environment through symbiosis with microorganisms (Chu et al., 2021). However, researches on the expression of genes related to N and K metabolism are mainly focused on rice roots, shoots, stems and leaves.

Previous studies have identified LOC_Os10g40600 as encoding a nitrate transporter NRT1.1B which has been demonstrated to mediate nitrate signal transduction. In this study, the expression level of *OsNRT1.1B* in rice grains increased as more N was gradually applied, while the impact of K application rate on *OsNRT1.1B* expression was limited (**Figure 5A**). These findings provided evidence that NRT1.1B expression was substantially induced by nitrate and was directly involved in nitrate uptake and nitrate transport (Hu et al., 2015). Ammonium uptake from the soil solution is mediated by ammonium transporters, such as the *OsAMT2;1* gene, which encodes a functional ammonium transporter and is a key gene in ammonium ion absorption pathway. It has been reported that the expression level of *OsAMT2;1* in rice cultivars with different N use efficiency varied under different N application rates. High N use efficiency rice cultivar showed higher gene expression levels in leaves under low N application levels (Yang, 2021). However, no obviously changes were observed in *OsAMT2;1* expression level in rice grains under different N and K application rates, except for N3K0, which requires further study (**Figure 5B**). Nitrate reductase (NR) is an important enzyme in plant N metabolism, playing a key role in N absorption and utilization. Bioinformatic analysis revealed two members of NR gene family in rice genome, NR1 and NR2, with over 70% similarity between their protein sequences (Zhao, 2010). During the grain filling process, NR activity in leaves first increased and then decreased. The activity of NR increased with increasing N levels (Lan, 2022). Consistent with this trend, the present study found that the expression level of *OsNR2* in rice grains increased with increasing N levels, and could be further enhanced by applying appropriate K application at high N levels (**Figure 5C**).

K plays irreplaceable roles in plant growth and development. The KUP/HAK/KT family, with the largest family members and diverse functions, is involved in nutrient metabolism, growth regulation and stress tolerance (Chai et al., 2019). Studies suggest that *OsHAK1*, *OsHAK5*, and *OsAKT1* are crucial for K^+^ uptake at both low and high concentrations, as well as for K^+^ translocation from root to shoot. *OsHAK1* and *OsAKT1* exhibit high expression levels in all cell types of roots and low levels in shoots (Manuel et al., 2016). Additionally, the transcription levels of HAK genes are regulated by the level of K nutrition. Overexpressing HAK1 or HAK5 in rice and maize increased K^+^ uptake, thereby increasing grain yield (Qin et al., 2019). In our studies, higher expression levels of *OsHAK1*, *OsHAK5*, and *OsAKT1* in rice grains were observed under N3K2 or N3K3, which were consistent with the above results of K uptake, indicating that a high N application level combined with a high K application level was beneficial to K absorption in rice (**Figures 5D–F**).

### 4.3 Effects of co-application N and K on rice yield and quality

Different N application patterns need to be combined with appropriate application of K to achieve optimal yields. Excessive application of either N or K not only results in fertilizer waste and environment pollution but also decrease fertilizer use efficiency (Wang et al., 2010b). Our results were corroborated with previous studies indicating that rice yield exhibited an incremental trend with increasing N application. However, beyond a certain threshold of N application, the yield increase became statistically insignificant (Hu et al., 2012). Over a two-year field experiment, our results demonstrated that co-application of N and K yielded a higher rice output compared to sole application of either N or K. These results indicated that appropriate combined application of N (180 kg ha^-1^) and K (135 kg ha^-1^) could not only improve GY but also reduced the fertilizer input. However, an increase in K application did not exhibit a pronounced effect on the improvement of yield at the same N level. Correlation analysis showed that GY was significantly positively correlated with PN and SP (**Figure 7**), indicating that the increase of PN and SP might be the primary factors contributing to the increased GY from N and K application (Yang and Zhang, 2011). Furthermore, the positive impact of K application on rice yield components might be attributed to its promotion of root development, subsequently enhancing nutrient uptake by plants. Despite N application leading to a decrease in seed setting rate (SR), K application had a counteractive effect, compensating for the overall grain yield formation. The decrease in SR might be attributed to the substantial enhancement of PN and SP induced by N application, expanding the capacity of “sink” rapidly. Nevertheless, the grain filling process could not be carried out or complete timely due to the limited supply of “source”, which in turn led to the decrease in SR (Sun et al., 2012).

It is widely reported that reasonable application of N can reduce CR and CD of rice, thus improving the appearance quality of rice (Zhang, 2017). And the appropriate application of K under conditions that meet the N requirements for rice growth can effectively increase HR and reduce CR and CD, thereby enhancing both the milling quality and appearance quality of rice (Zhang et al., 2018). Besides, the contribution rate affecting rice quality tended to be in the order of N > K > P, with the interactive effect of N and K surpassing that of N and P (Xie et al., 2014). In the present study, milling quality of YXY 2115 decreased with increasing N levels. As more K was gradually applied at the same N level, both MR and HR first increased and then decreased. The above results indicated that N and K mutually promoted each other, and their combined application was beneficial for improving the milling quality of rice. The appearance quality of YXY 2115 increased with increasing N levels. However, as more K was gradually applied at the same N level, obvious increases in CR and CD were found. This result was not consistent with the previous studies (Wang, 2015). The possible reason for this difference could be the relatively high content of available K in the experimental field soil, causing a decrease in the appearance quality of rice when additional K was applied. In general, higher fertilizer application tends to result in increased rice yield, but may reduce rice eating quality. Parts of rice quality indexes can be significantly enhanced within a certain level of K application (Ye, 2021). Co-application of N and K can increase protein content of rice, thereby improve rice nutritional quality. However, the lack of K will lead to a decline in both the eating quality and nutritional value of rice (Zhang, 2017). In our study, the cooking and eating quality of YXY 2115 decreased with increasing N levels. As more K was gradually applied at the same N level, the taste value, appearance and mouthfeel all first increased and then decreased, in agreement with the previous studies (Zhang and Qian, 2017). It is widely recognized that rice cultivars with high eating quality normally show higher peak viscosity and breakdown viscosity, coupled with lower setback viscosity, as determined by the RVA. Variance analysis showed that co-application of N and K had significant interactive effects on PV, BV and SV. However, no significant differences or prominent impacts were found in the response of RVA profile characters to co-application of N and K over a two-year field experiment, indicating that the combined application of N and K had limited effects on rice starch viscosity (Zhang, 2017). Taken together, in order to improve the appearance, milling, and cooking and eating quality of rice, a reasonable nitrogen-potassium ratio becomes particularly important. The optimal milling, cooking and eating quality were obtained under N1K2, while the best appearance quality was obtained under N3K0.

## 5 Conclusion

Reasonable combined application of N and K can mitigate fertilizer waste, reduce environmental pollution, lower production costs and increase economic benefits by improving fertilizer use efficiency, rice yield and quality, thereby achieving the sustainable development of agriculture. The present study investigated the effects of co-application of N and K on rice yield, quality and nutrients absorption and utilization through a two-year field experiment. The results revealed the feasibility of enhancing rice production efficiency and yield through co-application of N and K, which might provide an important reference for the large-scale promotion and application of Yixiangyou 2115. The highest rice yield was obtained under N2K2, while the optimal rice quality was obtained under N1K2. Thus, we recommended appropriate N application levels ranging from 206 kg ha^-1^ to 210 kg ha^-1^, combined with K application levels ranging from 115 kg ha^-1^ to 137 kg ha^-1^ as a preferable approach to achieve high yield for super hybrid indica rice cultivar Yixiangyou 2115 in southwest China. A combination of 135 kg ha^-1^ N and 135 kg ha^-1^ K application rates was recommended for achieving better quality in rice production.

## Data availability statement

The original contributions presented in the study are included in the article/**Supplementary Material**. Further inquiries can be directed to the corresponding authors.

## Author contributions

GC: Writing – original draft, Writing – review & editing. QD: Writing – review & editing, Writing – original draft. CW: Conceptualization, Writing – review & editing. XH: Investigation, Writing – review & editing. MH: Investigation, Writing – review & editing. CL: Data curation, Writing – review & editing. YO: Methodology, Writing – review & editing. LP: Formal analysis, Writing – review & editing. HY: Software, Writing – review & editing. QZ: Software, Writing – review & editing. QJ: Resources, Writing – review & editing. YL: Supervision, Validation, Writing – review & editing. TL: Funding acquisition, Project administration, Writing – review & editing.

## Supplementary material

Supplementary Table 1 Variance analysis of dry matter accumulation under different combined application of N and K.

Supplementary Table 2 Variance analysis of nutrients uptake under different combined application of N and K.

Supplementary Table 3 Effects of combined application of N and K on RVA profile characters of YXY 2115.

Supplementary Table 4 Effects of combined application of N and K on eating and cooking quality of YXY 2115.

Supplementary Table 5 Gene primers used for PCR amplification.
