# Supplementary material for: Optimizing rice yield, quality and nutrient use efficiency through combined application of nitrogen and potassium
Source: Front Plant Sci. 2024 Mar 11;15:1335744. doi: 10.3389/fpls.2024.1335744 (PMC10961459; doi:10.3389/fpls.2024.1335744)
Supplement: Supplementary Table 1 — Variance analysis of dry matter accumulation under different combined application of N and K. [file Table_1.docx]

**Table S1** Variance analysis of dry matter accumulation under different combined application of N and K.

| Growth stage | Treatment | Stem-sheath |  | Leaf |  | Panicle |  | Total |  |
| --- | --- | --- | --- | --- | --- | --- | --- | --- | --- |
|  |  | 2019 | 2020 | 2019 | 2020 | 2019 | 2020 | 2019 | 2020 |
| Full heading stage | N | 361.04** | 55.54** | 135.25** | 6.15** | 8.92** | 45.21** | 248.69** | 66.37** |
|  | K | 162.21** | 55.85** | 26.24** | 79.66** | 12.96** | 9.97** | 92.52** | 95.48** |
|  | N × K | 38.41** | 8.04** | 5.03** | 15.01** | 1.77 | 1.77 | 15.78** | 8.39** |
|  | Year | 28.53** |  | 19.68** |  | 2.70** |  | 23.60** |  |
| Maturity stage | N | 55.33** | 135.02** | 27.66** | 58.14** | 202.67** | 67.55** | 264.89** | 221.44** |
|  | K | 116.12** | 284.34** | 80.80** | 75.41** | 93.15** | 113.60** | 268.77** | 373.71** |
|  | N × K | 12.98** | 105.08** | 22.09** | 18.22** | 23.37** | 42.52** | 15.28** | 45.40** |
|  | Year | 27.26** |  | 3.20** |  | 40.10** |  | 27.09** |  |

N and K represent nitrogen and potassium, respectively. ANOVA *p* values and symbols were defined as: * *p* < 0.05; ** *p* < 0.01; ns: *p* > 0.05. The data presented are the mean ± standard deviation, *n* = 3.

**Table S2** Variance analysis of nutrients uptake under different combined application of N and K.

| Growth stage | Treatment | Stem-sheath |  | Leaf |  | Panicle |  | Total |  |
| --- | --- | --- | --- | --- | --- | --- | --- | --- | --- |
|  |  | 2019 | 2020 | 2019 | 2020 | 2019 | 2020 | 2019 | 2020 |
| N uptake at  full heading stage | N | 432.68** | 50.62** | 2579.11** | 75.68** | 36.05** | 313.92** | 3577.25** | 220.00** |
|  | K | 492.48** | 466.95** | 356.84** | 118.54** | 35.28** | 166.69** | 403.77** | 317.61** |
|  | N × K | 246.05** | 63.65** | 22.37** | 17.65** | 4.75** | 111.79** | 20.12** | 43.08** |
|  | Year | 205.39** |  | 140.33** |  | 46.60** |  | 110.83** |  |
| N uptake at  maturity stage | N | 221.83** | 445.45** | 4.30* | 134.63** | 220.99** | 42.80** | 243.34** | 360.32** |
|  | K | 317.02** | 130.61** | 17.74** | 52.71** | 54.75** | 48.57** | 123.67** | 171.50** |
|  | N × K | 155.73** | 61.58** | 78.97** | 14.30** | 17.90** | 10.98** | 34.73** | 12.00** |
|  | Year | 146.96** |  | 38.43** |  | 18.84** |  | 47.45** |  |
| K uptake at  full heading stage | N | 514.96** | 11.46** | 117.13** | 11.71** | 114.12** | 142.19** | 384.62** | 21.72** |
|  | K | 126.06** | 153.97** | 43.39** | 106.92** | 239.48** | 144.77** | 278.22** | 414.21** |
|  | N × K | 14.78** | 64.16** | 26.11** | 15.40** | 64.06** | 153.08** | 41.38** | 17.83** |
|  | Year | 59.76** |  | 29.72** |  | 106.15** |  | 54.44** |  |
| K uptake at  maturity stage | N | 115.18** | 5.51* | 4.36* | 0.50 | 325.95** | 135.75** | 222.97** | 82.52** |
|  | K | 59.66** | 87.51** | 25.92** | 19.75** | 118.99** | 242.66** | 111.68** | 220.57** |
|  | N × K | 89.19** | 58.01** | 47.93** | 84.60** | 149.64** | 190.91** | 38.89** | 86.91** |
|  | Year | 35.19** |  | 26.52** |  | 106.67** |  | 41.75** |  |

N and K represent nitrogen and potassium, respectively. ANOVA *p* values and symbols were defined as: * *p* < 0.05; ** *p* < 0.01; ns: *p* > 0.05. The data presented are the mean ± standard deviation, *n* = 3.

**Table S3-1** Effects of combined application of N and K on RVA profile characters of YXY 2115 in 2019.

| Year | Treatment | | PV (RVU) | TV (RVU) | BV (RVU) | FV (RVU) | SV (RVU) | PeT (min) | PaT (℃) |
| --- | --- | --- | --- | --- | --- | --- | --- | --- | --- |
| 2019 | N1 | K0 | 248.46±3.80ef | 132.13±2.44e | 116.33±1.36bc | 222.92±3.55de | -25.54±0.27e | 5.93±0.08abc | 71.68±0.81cd |
|  |  | K1 | 261.67±6.50b | 139.71±1.42c | 121.96±7.78ab | 237.96±4.85ab | -23.71±2.15de | 5.93±0.07abc | 70.85±0.39f |
|  |  | K2 | 255.38±2.30bcd | 130.71±0.67e | 124.67±1.99a | 222.75±2.77de | -32.63±1.96f | 5.80±0.11c | 71.88±0.40bcd |
|  |  | K3 | 251.71±2.96cde | 142.75±1.55b | 108.96±4.47de | 237.42±9.18ab | -14.29±3.53b | 6.03±0.08a | 72.35±0.28b |
|  |  | Mean | 254.31A | 136.33A | 117.98A | 230.26A | -24.04B | 5.92A | 71.69B |
|  | N2 | K0 | 257.46±1.55bc | 130.71±0.65e | 126.75±0.91a | 224.17±3.76de | -33.29±4.01f | 5.80±0.06c | 71.14±0.07ef |
|  |  | K1 | 270.17±3.68a | 146.25±1.26a | 123.92±2.42a | 242.13±3.84a | -28.04±2.00ef | 6.00±0.12ab | 71.44±0.05de |
|  |  | K2 | 242.46±1.79fg | 130.46±1.43e | 112.00±1.66cd | 224.50±3.59de | -17.96±2.72bc | 5.87±0.01bc | 71.44±0.11de |
|  |  | K3 | 232.96±4.93h | 125.71±1.83f | 107.25±3.19de | 218.54±1.81e | -14.42±3.37b | 5.90±0.06abc | 71.90±0.04bcd |
|  |  | Mean | 250.76B | 133.28B | 117.48A | 227.34AB | -23.43B | 5.89A | 71.48B |
|  | N3 | K0 | 249.67±1.38de | 136.58±0.95d | 113.08±2.30cd | 229.58±2.03cd | -20.08±3.36cd | 5.97±0.04ab | 72.25±0.10b |
|  |  | K1 | 252.38±0.07cde | 131.08±0.22e | 121.29±0.16ab | 220.71±2.73e | -31.67±2.74f | 5.93±0.03abc | 72.03±0.03bc |
|  |  | K2 | 239.13±2.67g | 135.54±2.05d | 103.58±0.75e | 233.13±5.30bc | -6.00±2.63a | 5.90±0.07abc | 71.86±0.19bcd |
|  |  | K3 | 232.46±4.66h | 126.58±1.79f | 105.88±2.88e | 219.46±0.69e | -13.00±4.07b | 5.90±0.04abc | 72.85±0.11a |
|  |  | Mean | 243.41C | 132.45B | 110.96B | 225.72B | -17.69A | 5.93A | 72.25A |
|  | N |  | 30.69** | 22.62** | 18.41** | 3.58* | 17.31** | 0.77 | 20.17** |
|  | K |  | 67.43** | 46.61** | 38.49** | 7.89** | 44.90** | 3.47* | 15.00** |
|  | N × K |  | 14.37** | 68.23** | 10.65** | 14.88** | 22.25** | 3.24* | 3.37* |

N1, N2 and N3 refer to the different nitrogen fertilizer treatments (135, 180 and 225 kg ha^-1^, respectively). K0, K1, K2 and K3 refer to the different potassium fertilizer treatments (0, 90, 135 and 180 kg ha_-1_, respectively). PV, TV, BV, FV, SV, PeT and PaT represent peak viscosity, trough viscosity, breakdown viscosity, final viscosity, setback viscosity, peak time and pasting temperature, respectively. Different lowercase letters followed the values in the same column mean the significant difference of the different combined application of N and K levels at *p* < 0.05. Different uppercase letters mean the significant difference of different average N levels at *p* < 0.05. ANOVA *p* values and symbols were defined as: * *p* < 0.05; ** *p* < 0.01; ns: *p* > 0.05. The data presented are the mean ± standard deviation, *n* = 3.

**Table S3-2** Effects of combined application of N and K on RVA profile characters of YXY 2115 in 2020.

| Year | Treatment | | PV (RVU) | TV (RVU) | BV (RVU) | FV (RVU) | SV (RVU) | PeT (min) | PaT (℃) |
| --- | --- | --- | --- | --- | --- | --- | --- | --- | --- |
| 2020 | N1 | K0 | 158.83±4.38g | 98.25±0.16g | 60.58±4.23g | 179.67±3.47i | 20.83±7.78a | 6.13±0.04cd | 72.85±0.43ab |
|  |  | K1 | 240.92±0.60b | 139.58±1.27b | 101.33±0.67ab | 221.67±2.49b | -19.25±3.09de | 6.20±0.03bc | 72.10±1.89ab |
|  |  | K2 | 255.75±8.31a | 149.42±2.31a | 106.33±3.05a | 231.33±4.92a | -24.42±3.43e | 6.27±0.03ab | 71.25±1.29b |
|  |  | K3 | 228.33±4.90c | 135.58±2.19c | 92.75±2.78cd | 216.08±2.14cd | -12.25±6.15cd | 6.27±0.07ab | 72.60±0.28ab |
|  |  | Mean | 220.96A | 130.71A | 90.25B | 212.19A | -8.77AB | 6.22A | 72.20A |
|  | N2 | K0 | 221.08±0.77de | 126.50±2.10e | 94.58±2.26c | 207.67±2.82fg | -13.42±2.29cd | 6.13±0.03cd | 72.53±0.08ab |
|  |  | K1 | 226.25±2.56cd | 128.50±1.02de | 97.75±3.49bc | 214.17±1.52de | -12.08±3.99cd | 6.07±0.03d | 72.65±0.78ab |
|  |  | K2 | 235.33±2.72b | 130.67±0.62d | 104.67±2.11a | 216.42±2.49cd | -18.92±0.93de | 6.07±0.03d | 72.10±0.19ab |
|  |  | K3 | 215.25±1.13e | 127.08±1.61e | 88.17±0.94de | 212.58±4.21def | -2.67±4.24b | 6.07±0.03d | 72.65±0.43ab |
|  |  | Mean | 224.48A | 128.19B | 96.29A | 212.71A | -11.77B | 6.09B | 72.48A |
|  | N3 | K0 | 202.83±2.64f | 123.42±0.98f | 79.42±2.23f | 202.17±2.49h | -0.67±1.20b | 6.20±0.12bc | 72.78±0.18ab |
|  |  | K1 | 221.75±1.67cde | 127.58±1.34e | 94.17±2.79c | 209.50±2.21efg | -12.25±3.70cd | 6.20±0.12bc | 72.20±0.13ab |
|  |  | K2 | 226.08±2.01cd | 139.67±2.21b | 86.42±2.68e | 219.58±2.15bc | -6.50±2.71bc | 6.33±0.04a | 72.85±1.23ab |
|  |  | K3 | 206.75±2.85f | 127.08±2.05e | 79.67±4.88f | 207.00±0.39gh | 0.25±2.51b | 6.20±0.08bc | 73.30±0.16a |
|  |  | Mean | 214.35B | 129.44AB | 84.92C | 209.56B | -4.79A | 6.23A | 72.78A |
|  | N |  | 25.32** | 7.14** | 45.84** | 4.23* | 9.36** | 19.61** | 1.54 |
|  | K |  | 270.59** | 331.90** | 103.32** | 132.87** | 44.10** | 2.33 | 1.78 |
|  | N × K |  | 109.19** | 146.35** | 40.06** | 40.73** | 25.12** | 2.42 | 0.86 |
|  | Year |  | 67.66** | 102.61** | 24.03** | 19.55** | 20.80** | 3.96** | 1.33 |

N1, N2 and N3 refer to the different nitrogen fertilizer treatments (135, 180 and 225 kg ha^-1^, respectively). K0, K1, K2 and K3 refer to the different potassium fertilizer treatments (0, 90, 135 and 180 kg ha_-1_, respectively). PV, TV, BV, FV, SV, PeT and PaT represent peak viscosity, trough viscosity, breakdown viscosity, final viscosity, setback viscosity, peak time and pasting temperature, respectively. Different lowercase letters followed the values in the same column mean the significant difference of the different combined application of N and K levels at *p* < 0.05. Different uppercase letters mean the significant difference of different average N levels at *p* < 0.05. ANOVA *p* values and symbols were defined as: * *p* < 0.05; ** *p* < 0.01; ns: *p* > 0.05. The data presented are the mean ± standard deviation, *n* = 3.

**Table S4-1** Effects of combined application of N and K on eating and cooking quality of YXY 2115 in 2019.

| Year | Treatment | | Taste value | Appearance | Mouthfeel | Hardness | Stickiness | Balance | Flexibility value |
| --- | --- | --- | --- | --- | --- | --- | --- | --- | --- |
| 2019 | N1 | K0 | 86.00±0.00bc | 8.41±0.02bcd | 7.41±0.14d | 2.25±0.04h | 0.25±0.01cde | 0.11±0.05ab | 0.89±0.02a |
|  |  | K1 | 86.33±0.58b | 8.52±0.23ab | 8.02±0.21ab | 2.93±0.03cde | 0.24±0.04de | 0.08±0.01b | 0.88±0.03a |
|  |  | K2 | 88.67±0.58a | 8.70±0.10a | 8.22±0.18a | 2.30±0.07h | 0.22±0.02e | 0.10±0.04ab | 0.89±0.03a |
|  |  | K3 | 86.33±0.58b | 8.62±0.19ab | 7.91±0.37abc | 3.17±0.21b | 0.27±0.01bcd | 0.09±0.04ab | 0.89±0.08a |
|  |  | Mean | 86.83A | 8.56A | 7.89A | 2.66B | 0.25B | 0.10A | 0.89A |
|  | N2 | K0 | 83.00±1.00d | 8.15±0.08de | 7.54±0.08cd | 2.80±0.12def | 0.24±0.01de | 0.09±0.01ab | 0.88±0.04a |
|  |  | K1 | 84.33±1.15cd | 8.40±0.07bcd | 7.63±0.04bcd | 3.14±0.12bc | 0.22±0.04e | 0.07±0.01b | 0.87±0.01a |
|  |  | K2 | 86.00±1.00bc | 8.63±0.10ab | 7.70±0.02bcd | 2.73±0.12efg | 0.26±0.04cde | 0.10±0.03ab | 0.85±0.04a |
|  |  | K3 | 85.67±1.53bc | 8.44±0.18abc | 7.70±0.03bcd | 2.52±0.22g | 0.34±0.03a | 0.13±0.06a | 0.90±0.01a |
|  |  | Mean | 84.75B | 8.41A | 7.64B | 2.80B | 0.27A | 0.10A | 0.88A |
|  | N3 | K0 | 83.00±1.00d | 8.21±0.27cde | 7.53±0.08cd | 3.01±0.16bcd | 0.31±0.04ab | 0.10±0.03ab | 0.88±0.01a |
|  |  | K1 | 84.33±0.58cd | 8.22±0.33cde | 7.61±0.03cd | 2.58±0.28fg | 0.23±0.04de | 0.09±0.03ab | 0.87±0.01a |
|  |  | K2 | 82.67±0.58d | 8.00±0.05e | 7.42±0.14d | 3.61±0.08a | 0.29±0.01bc | 0.08±0.01b | 0.86±0.01a |
|  |  | K3 | 79.00±2.65e | 7.49±0.07f | 7.02±0.54e | 3.62±0.18a | 0.26±0.03cde | 0.07±0.01b | 0.86±0.03a |
|  |  | Mean | 82.25C | 7.98B | 7.40C | 3.21A | 0.27A | 0.09A | 0.87A |
|  | N |  | 38.52** | 15.54** | 49.46** | 39.97** | 3.16 | 0.54 | 1.18 |
|  | K |  | 4.40* | 4.01* | 6.52** | 10.90** | 7.25** | 0.71 | 0.58 |
|  | N × K |  | 6.91** | 4.12** | 6.89** | 27.84** | 5.03** | 1.12 | 0.50 |
|  | Year |  |  |  |  |  |  |  |  |

N1, N2 and N3 refer to the different nitrogen fertilizer treatments (135, 180 and 225 kg ha^-1^, respectively). K0, K1, K2 and K3 refer to the different potassium fertilizer treatments (0, 90, 135 and 180 kg ha_-1_, respectively). Different lowercase letters followed the values in the same column mean the significant difference of the different combined application of N and K levels at *p* < 0.05. Different uppercase letters mean the significant difference of different average N levels at *p* < 0.05. ANOVA *p* values and symbols were defined as: * *p* < 0.05; ** *p* < 0.01; ns: *p* > 0.05. The data presented are the mean ± standard deviation, *n* = 3.

**Table S4-2** Effects of combined application of N and K on eating and cooking quality of YXY 2115 in 2020.

| Year | Treatment | | Taste value | Appearance | Mouthfeel | Hardness | Stickiness | Balance | Flexibility value |
| --- | --- | --- | --- | --- | --- | --- | --- | --- | --- |
| 2020 | N1 | K0 | 80.00±1.73f | 7.63±0.09f | 6.93±0.05f | 2.37±0.02e | 0.29±0.13defg | 0.12±0.04efg | 0.88±0.01abc |
|  |  | K1 | 87.33±1.53abc | 8.90±0.03ab | 8.31±0.04ab | 1.88±0.03g | 0.43±0.05abc | 0.23±0.03a | 0.90±0.04ab |
|  |  | K2 | 89.00±0.00a | 9.01±0.11a | 8.64±0.11a | 2.34±0.02e | 0.35±0.1bcde | 0.15±0.04de | 0.89±0.04abc |
|  |  | K3 | 88.33±1.53ab | 8.81±0.23abc | 8.02±0.83bc | 1.72±0.05h | 0.33±0.04cdef | 0.19±0.02bc | 0.90±0.04ab |
|  |  | Mean | 86.17A | 8.59A | 7.98A | 2.08C | 0.35A | 0.17A | 0.89A |
|  | N2 | K0 | 83.33±1.15e | 8.11±0.08e | 7.51±0.14de | 2.80±0.05b | 0.24±0.09fg | 0.09±0.03gh | 0.88±0.01abc |
|  |  | K1 | 84.33±0.58de | 8.42±0.11d | 7.60±0.09cde | 3.14±0.12a | 0.22±0.05g | 0.07±0.03h | 0.87±0.03bc |
|  |  | K2 | 86.00±1.00cd | 8.64±0.23c | 7.75±0.09cde | 2.73±0.04bc | 0.26±0.05efg | 0.10±0.04fgh | 0.85±0.03c |
|  |  | K3 | 85.67±0.58cd | 8.40±0.08d | 7.73±0.14cde | 2.52±0.04d | 0.34±0.05cdef | 0.13±0.04ef | 0.90±0.01ab |
|  |  | Mean | 84.83B | 8.39B | 7.65B | 2.80A | 0.27B | 0.10B | 0.88B |
|  | N3 | K0 | 80.67±0.58f | 7.96±0.10e | 6.88±0.23f | 1.78±0.08gh | 0.37±0.04abcd | 0.21±0.04ab | 0.92±0.01a |
|  |  | K1 | 87.00±1.00bc | 8.75±0.04bc | 7.82±0.03cd | 2.11±0.08f | 0.36±0.04bcde | 0.17±0.02cd | 0.89±0.01abc |
|  |  | K2 | 84.33±0.58de | 8.40±0.09d | 7.63±0.02cde | 2.51±0.15d | 0.45±0.05ab | 0.18±0.01bcd | 0.86±0.01bc |
|  |  | K3 | 83.67±0.58e | 8.13±0.10e | 7.29±0.19ef | 2.63±0.06cd | 0.47±0.05a | 0.18±0.02bcd | 0.89±0.01abc |
|  |  | Mean | 83.92B | 8.31B | 7.41C | 2.26B | 0.41A | 0.19A | 0.89A |
|  | N |  | 16.29** | 14.02** | 14.55** | 333.63** | 14.35** | 29.32** | 2.15 |
|  | K |  | 82.83** | 20.92** | 50.62** | 19.97** | 2.20 | 1.49 | 3.25* |
|  | N × K |  | 16.78** | 5.24** | 11.15** | 86.30** | 1.95 | 4.52** | 1.75 |
|  | Year |  | 10.38** | 2.51* | 8.97** | 37.74** | 3.49** | 4.86** | 0.44 |

N1, N2 and N3 refer to the different nitrogen fertilizer treatments (135, 180 and 225 kg ha^-1^, respectively). K0, K1, K2 and K3 refer to the different potassium fertilizer treatments (0, 90, 135 and 180 kg ha_-1_, respectively). Different lowercase letters followed the values in the same column mean the significant difference of the different combined application of N and K levels at *p* < 0.05. Different uppercase letters mean the significant difference of different average N levels at *p* < 0.05. ANOVA *p* values and symbols were defined as: * *p* < 0.05; ** *p* < 0.01; ns: *p* > 0.05. The data presented are the mean ± standard deviation, *n* = 3.

**Table S5** Gene primers used for PCR amplification.

| Genes | MSU Locus | Purpose | Forward primer (5’-3’) | Reverse primer (5’-3’) |
| --- | --- | --- | --- | --- |
| *OsNRT1.1B* | LOC_Os10g40600 | Q-PCR | GGCAGGCTCGACTACTTCTA | AGGCGCTTCTCCTTGTAGAC |
| *OsAMT2;1* | LOC_Os05g39240 | Q-PCR | GATGAATCACGCCGAAACAC | GCACGGACGAATCGCTACTT |
| *OsNR2* | LOC_Os02g53130 | Q-PCR | ACTACCATTACCGCGACAACCG | TGTACCACCAAGCTTCGGCATTG |
| *OsHAK1* | LOC_Os04g32920 | Q-PCR | GTTGATGATGCTGATGTTGGAAG | CCAACACTTTCAGCTGAAAC |
| *OsHAK5* | LOC_Os01g70490 | Q-PCR | CATTGTGGACTATTTTGAAAGAA | GGAGAACTACAGAAAGCCAATC |
| *OsAKT1* | LOC_Os01g45990 | Q-PCR | TTGCGTTTGAATCGTACAGC | CCTTTACGACACCAGCCATT |
| *OsACTIN* |  | Q-PCR | TGGCATCTCTCAGCACATTCC | TGCACAATGGATGGGTCAGA |
